# Evaluation of Aminoglycoside Dosing Regimens Adjusted for Renal Function and In Vitro Susceptibility Test Interpretive Criteria for Enterobacterales and *Pseudomonas aeruginosa*

**DOI:** 10.1093/ofid/ofaf426

**Published:** 2025-11-26

**Authors:** Sujata M Bhavnani, Jeffrey P Hammel, Nikolas J Onufrak, Jason M Pogue, Ronald N Jones, Helio S Sader, George L Drusano, John S Bradley, David R Andes, William A Craig, Paul G Ambrose

**Affiliations:** Institute for Clinical Pharmacodynamics, Inc., Schenectady, New York, USA; Institute for Clinical Pharmacodynamics, Inc., Schenectady, New York, USA; Institute for Clinical Pharmacodynamics, Inc., Schenectady, New York, USA; Department of Clinical Pharmacy, College of Pharmacy, University of Michigan, Ann Arbor, Michigan, USA; JMI Laboratories/Element, North Liberty, Iowa, USA; JMI Laboratories/Element, North Liberty, Iowa, USA; Institute for Therapeutic Innovation, University of Florida, Orlando, Florida, USA; University of California at San Diego, San Diego, California, USA; Rady Children's Hospital, San Diego, California, USA; Departments of Medicine and Medical Microbiology and Immunology, University of Wisconsin-Madison, Madison, Wisconsin, USA; Department of Medicine, University of Wisconsin-Madison School of Medicine and Public Health, Madison, Wisconsin, USA; Institute for Clinical Pharmacodynamics, Inc., Schenectady, New York, USA

**Keywords:** aminoglycosides, in vitro susceptibility test interpretive criteria, susceptibility breakpoints

## Abstract

**Background:**

Appropriate in vitro susceptibility testing interpretive criteria (STIC) are crucial to ensure optimal patient care. Pharmacometric analyses for aminoglycosides were undertaken to provide recommendations for dosing regimens adjusted for renal function and to support the original 2019 and 2023 reassessments of STIC for Enterobacterales and *Pseudomonas aeruginosa*.

**Methods:**

Using simulation, traditional and extended-interval aminoglycoside dosing regimens adjusted for renal function that match drug exposures for patients with normal renal function were constructed. Using in vitro surveillance, nonclinical pharmacokinetic–pharmacodynamic (PK-PD), population pharmacokinetic (PK) model data, and simulation, PK-PD target attainment analyses were carried out to assess aminoglycoside STIC for Enterobacterales and *P. aeruginosa*. Susceptible breakpoints identified represented the highest minimum inhibitory concentration (MIC) values at which percent probabilities of PK-PD target attainment approached or were ≥90% based upon area under the concentration-time curve (AUC):MIC ratio targets associated with a 1-log_10_ colony-forming unit reduction from baseline at 24 hours, and extended-interval dosing regimens. Susceptible breakpoints based on therapeutic drug monitoring (S-TDM) were also considered as such data can be used to adjust dose to achieve targeted AUC values for efficacy, in addition to monitoring for safety.

**Results:**

Aminoglycoside dosing regimens adjusted for renal function were successfully identified. For Enterobacterales and *P. aeruginosa*, recommended susceptible, S-TDM, and resistance breakpoints were ≤2, 4, and ≥8 µg/mL, respectively, for amikacin and ≤0.5, 1, and ≥2 µg/mL, respectively, for gentamicin and tobramycin, based on the evaluation of extended-interval dosing regimens.

**Conclusions:**

Use of dosing recommendations adjusted for renal function and United States Committee on Antimicrobial Susceptibility Testing–recommended STIC will allow clinicians to optimally use aminoglycosides.

## Teaser

The results of pharmacometric analyses for aminoglycosides were used to support recommendations for dosing regimens adjusted for renal function and a reassessment of the USCAST STIC for Enterobacterales and Pseudomonas aeruginosa. Susceptible and susceptible based on therapeutic drug monitoring breakpoints are recommended for extended interval amikacin, gentamicin, and tobramycin dosing regimens.

The threat arising from infections caused by antimicrobial-resistant bacteria continues to be of global concern [1, 2]. Antibiotic misuse is a major driver of antimicrobial resistance [3]. The use of inappropriate in vitro susceptibility testing interpretive criteria (STIC) for antimicrobial agents can lead to misuse through suboptimal treatment choices [4]. The re-evaluation of STIC using a modern approach, which includes evaluating the results of pharmacometric analyses, represents an important mechanism to improve the understanding of antimicrobial resistance in order to select the most optimal antimicrobial regimens to improve patient outcomes. Appropriate STIC ensure that older agents are assessed relative to new standards. This allows for better characterization of the prevalence of antimicrobial resistance among agents that are chosen for empiric therapy and optimization of antimicrobial therapy for individual patients based on STIC for recovered pathogens [4].

A pharmacometric approach for assessing STIC is well established and involves the use of in vitro surveillance data, pharmacokinetic–pharmacodynamic (PK-PD) data from nonclinical infection models, population pharmacokinetic (PK) models, and simulation to carry out PK-PD target attainment analyses [5–7]. The results from such an approach are particularly helpful when clinical data describing the percentage of patients with clinical or microbiological success by minimum inhibitory concentration (MIC) are limited. When available, clinical data are often limited by the retrospective design of available studies and the subsequent biases associated with such methodology (eg, confounding by indication), small sample sizes, the historic rather than contemporary nature of dosing strategies employed, the use of ill-defined combination therapy, and the lack of detailed information about how efficacy endpoints were assessed.

Aminoglycosides represent an older class of antimicrobial agents for which the STIC required re-evaluation. Gentamicin was approved in 1964, followed by tobramycin in 1975, and amikacin in 1976 [8–10]. STIC for aminoglycosides were established by the Clinical and Laboratory Standards Institute (CLSI), then known as the National Committee for Clinical Laboratory Standards (NCCLS), more than 4 decades ago [11]. At that time, STIC were largely informed by the shape of MIC distributions, specifically, wild-type MIC distributions. As described by Barry et al. in 1981 [12], while the pharmacokinetics of amikacin and gentamicin were considered, definitions for resistant breakpoints were guided by concentration thresholds for toxicity. Since this time, the PK-PD for the efficacy and safety of aminoglycosides have been well characterized, and as a result, aminoglycosides are administered in larger doses with an extended interval to optimize efficacy and safety [13, 14].

In 2014, the United States Committee on Antimicrobial Susceptibility Testing (USCAST) began the process to evaluate the aminoglycoside STIC. This evaluation considered STIC for amikacin, gentamicin, and tobramycin against Enterobacterales and *P. aeruginosa* using the above-described pharmacometric approach in the context of combination therapy and infection site [15, 16] and a PK-PD target associated with net bacterial stasis. Given that existing package inserts for amikacin, gentamicin, and tobramycin do not provide guidance for extended-interval dosing regimens and that there is limited guidance for dose adjustments for renal impairment and augmented renal function [17–19], an additional objective of these analyses was to develop such dosing regimen recommendations for these agents. Herein, we describe the results of pharmacometric analyses, which include recommendations for aminoglycoside dosing regimens adjusted for renal function and PK-PD target attainment analyses that were used to support the original 2019 [15, 16] and the recent reassessment of aminoglycoside STIC for amikacin, gentamicin, and tobramycin against Enterobacterales and *P. aeruginosa*.

## METHODS

### In Vitro Surveillance Data

MIC distributions for amikacin, gentamicin, and tobramycin against 47130 Enterobacterales and 8906 *P. aeruginosa* isolates collected in the United States from 2017 to 2021 as part of the SENTRY Antimicrobial Surveillance Program (JMI Laboratories, North Liberty, IA, USA) were used to interpret the results of the PK-PD target attainment analyses. Antimicrobial susceptibility was evaluated by broth microdilution method according to CLSI procedures [20]. MIC distributions for the subsets of Enterobacterales isolates that demonstrated an extended-spectrum beta-lactamase (ESBL) phenotype, that were multidrug-resistant (MDR), or carbapenem-resistant (CRE) were also evaluated. ESBL phenotype was defined as follows: *Escherichia coli*, *Klebsiella pneumoniae*, *Klebsiella oxytoca*, and *Proteus mirabilis* isolates displaying MIC values ≥2 µg/mL for ceftazidime, ceftriaxone, or aztreonam [21]. Isolates were considered MDR if they were nonsusceptible to ≥1 antimicrobial agent in ≥3 antimicrobial classes [22]. If isolates had imipenem and/or meropenem MIC values >2 µg/mL, these were considered CRE [21]. Imipenem results were not used to classify *P. mirabilis* or indole-positive *Proteus*. Epidemiological cutoff (ECOFF) values were calculated as described by the European Committee on Antimicrobial Susceptibility Testing (EUCAST) [23, 24]. Calculations of ECOFF values were based on data for all isolates in the collections for each of Enterobacterales and *P. aeruginosa*.

### Nonclinical Pharmacokinetic–Pharmacodynamic Studies

Data from previous dose-fractionation studies conducted using a neutropenic murine-thigh infection model served to demonstrate that the ratio of the area under the concentration-time curve (AUC) to the MIC (AUC:MIC ratio) was the PK-PD index most predictive of efficacy for aminoglycosides [25]. The magnitude of AUC:MIC ratio targets associated with various levels of bacterial reduction for amikacin, gentamicin, and tobramycin against Enterobacterales and *P. aeruginosa* were derived from the results of dose-ranging studies that were carried out using murine-thigh and -lung infection models and PK-PD analyses of these data. A brief description of the methods of these studies is described in the Supplementary Data.

Using the data from the above-described dose-ranging studies, the relationships between log_10_ colony-forming unit (CFU) reduction from baseline at 24 hours and total-drug plasma or total-drug epithelial lining fluid (ELF) AUC:MIC ratio were evaluated using Hill-type models and nonlinear least-squares regression by pathogen or pathogen group. The magnitudes of the AUC:MIC ratio associated with net bacterial stasis and a 1-log_10_ CFU reduction from baseline for Enterobacterales and *P. aeruginosa*, which represented endpoints of interest for the PK-PD target attainment analyses, were determined. As described in the Supplementary Data, the binding of aminoglycosides to plasma proteins, including in mice, is low. Thus, no corrections were made for total-drug plasma AUC:MIC ratio targets by agent, and this served as the basis for pooling data for these 3 agents when evaluating the above-described PK-PD relationships for efficacy.

### Simulated Patient Data

PK-PD target attainment analyses were carried out using data from simulated patients that were generated using R, version 3.3.1 [26]. To form the simulated population, 1000 patients were chosen randomly and with replacement from patients in a pooled collection of clinical trials [27]. The simulated patient population thus had distributions of demographic data representative of the pooled clinical trial population. The same simulated patient population was evaluated 6 times, assessing baseline creatinine clearance (CLcr, mL/min) that was assigned using a uniform probability distribution for each of the following 6 CLcr groups: ≥16 to ≤30, >30 to ≤45, >45 to ≤60, >60 to ≤90, >90 to ≤120, and >120 to ≤240 mL/min. Plasma concentration-time profiles were generated for each simulated patient after administration of dosing regimens for each agent described below using an appropriate population PK model. Population PK models for each agent from the published literature were selected to carry out simulations. Further details about the simulations, the criteria used to discriminate among candidate population PK models, and the process to generate individual PK parameter values for each agent among simulated patients are described in the Supplementary Data.

As described in the Supplementary Data, the development of traditional and extended-interval aminoglycoside weight-based dosing regimens administered to simulated patients in the above-described 6 CLcr groups was guided by those described in the United States Food and Drug Administration (FDA) package inserts [17–19] and those commonly recommended among hospitals, as summarized in Supplementary Tables 1 and 2, respectively. Traditional or extended-interval dosing regimens for each agent identified for a given CLcr group were selected to provide matching total-drug plasma AUC and minimum concentration (C_min_) values for dosing regimens administered to simulated patients with normal renal function (ie, CLcr >90 to ≤120 mL/min). The specific criteria used to select dosing regimens by CLcr group are described in the Supplementary Data.

Total-drug plasma and total-drug ELF AUC values from 0 to 48 hours (AUC_0–48_) for each agent were calculated using numerical integration. The average 24-hour AUC over this period (AUC_0–24_), which was calculated by dividing the AUC_0–48_ in half, was used to compare AUC values among simulated patients receiving dosing regimens from every 8 hours (q8h) to every 48 hours (q48h). As demonstrated by protein-binding point estimates for each agent, shown in Supplementary Table 3 [17–19], and low magnitude of protein binding and dependence of results on test conditions as described in the Supplementary Data, PK-PD target attainment analyses were based on total-drug plasma rather than free-drug plasma AUC values. As described in the Supplementary Data, average total-drug ELF AUC_0–24_ values over 48 hours were generated for simulated patients by multiplying average total-drug plasma AUC_0–24_ values by an ELF penetration ratio [28, 29].

### Pharmacokinetic–Pharmacodynamic Target Attainment Analyses

Using the average total-drug plasma and total-drug ELF AUC 0-24 values over 48 hours, percent probabilities of PK-PD target attainment by MIC among simulated patients by CLcr group after administration of traditional and extended-interval aminoglycoside dosing regimens for each agent were calculated. Total-drug plasma AUC:MIC ratio targets associated with net bacterial stasis and a 1-log_10_ CFU reduction from baseline for aminoglycosides against Enterobacterales and *P. aeruginosa* derived using a neutropenic murine-thigh infection model were assessed. Total-drug ELF AUC:MIC ratio targets associated with the same endpoints for aminoglycosides against Enterobacterales determined using a neutropenic murine-lung infection model were also assessed. Given that no such studies for *P. aeruginosa* using a neutropenic murine-lung infection model were carried out, total-drug ELF AUC:MIC ratio targets associated with these endpoints could not be determined or assessed as part of these analyses.

Percent probabilities of PK-PD target attainment by MIC were determined using 3 approaches for selecting AUC:MIC ratio targets associated with net bacterial stasis and a 1-log_10_ CFU reduction from baseline for Enterobacterales and *P. aeruginosa*. These included AUC:MIC ratio targets based on Hill-type models constructed using pooled data from all isolates by pathogen or pathogen group and both median and randomly assigned AUC:MIC ratio targets. Randomly assigned AUC:MIC ratio targets were based on an estimated log normal distribution of AUC:MIC ratio targets associated with a given endpoint. As described previously [30], distributions of AUC:MIC ratio targets were truncated on the log scale such that randomly selected targets were all within 2 standard deviations of the mean value. The results of PK-PD target attainment analyses were interpreted in the context of in vitro surveillance data for Enterobacterales, including subsets of ESBL phenotype, MDR, and CRE isolates (ie, the resistant subsets), and *P. aeruginosa*.

### Susceptibility Testing Interpretive Criteria Assessments

The results of PK-PD target attainment analyses for extended-interval aminoglycoside dosing were used for the assessment of STIC for Enterobacterales and *P. aeruginosa* as extended-interval dosing is considered safer and is guideline-recommended for aminoglycosides when treating patients with serious gram-negative infections [31–33]. Susceptible breakpoints identified for each agent against Enterobacterales and *P. aeruginosa* represented the highest MIC value at which the percent probabilities of PK-PD target attainment approached or were ≥90% based upon AUC:MIC ratio targets associated with a 1-log_10_ CFU reduction from baseline. Given that dosing regimens were selected to achieve similar drug exposures among simulated patients in all CLcr groups relative to the normal renal function group, CLcr >90 to ≤120 mL/min, STIC recommendations based only on data for simulated patients with normal renal function were considered sufficient.

Susceptible breakpoints based on therapeutic drug monitoring (S-TDM) were also considered given the expectation that therapeutic drug monitoring (TDM) is carried out in most medical centers in the United States and that clinicians use trough concentrations to monitor for safety and adjust aminoglycoside dosing regimens as needed. The premise for S-TDM breakpoints is that while empiric aminoglycoside dosing regimens cannot reliably achieve the AUC:MIC ratio targets associated with a 1-log_10_ CFU reduction from baseline at the MIC values representing the susceptible breakpoints in all patients, TDM data can be used by clinicians to adjust subsequent doses while considering safety in order to achieve the above-described AUC:MIC ratio targets for a given MIC value. Using an individualized approach, higher susceptible breakpoints based on TDM (ie, S-TDM) may be possible and thus, were considered. However, for institutions for which this is not possible, isolates with MIC values greater than the susceptible breakpoint should be considered nonsusceptible.

## RESULTS

### In Vitro Surveillance Data

Aminoglycoside MIC distributions for Enterobacterales, including subsets of ESBL phenotype, MDR, and CRE isolates, and *P. aeruginosa* are shown in Supplementary Tables 4 and 5, respectively. The aminoglycoside ECOFF values for Enterobacterales and *P. aeruginosa* isolates, which were calculated using all isolates in the collection, are shown in Supplementary Table 6.

### Nonclinical Pharmacokinetic–Pharmacodynamic Studies

**Figure 1.**
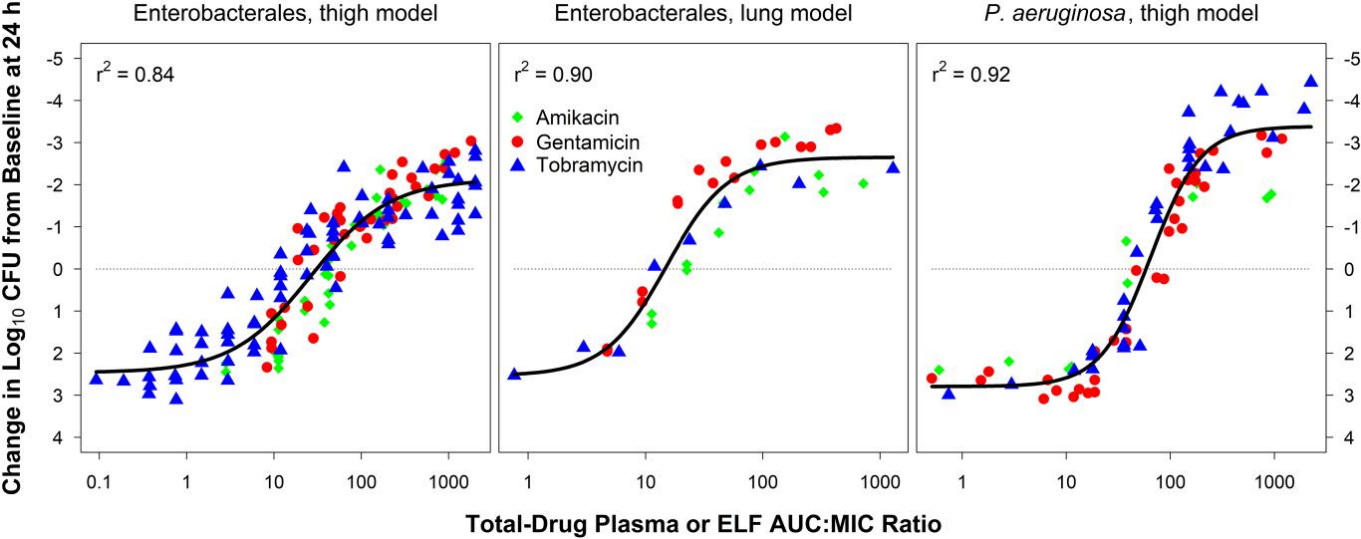
Relationships between change in log_10_ CFU from baseline of Enterobacterales in the thighs and lungs and *P. aeruginosa* in the thighs of neutropenic mice after 24 hours of therapy with aminoglycosides and each of total-drug plasma and total-drug ELF AUC:MIC ratio based on data from neutropenic murine-thigh and -lung infection models, respectively. Abbreviations: AUC, area under the concentration-time curve; AUC: MIC ratio, ratio of the area under the concentration-time curve to minimum inhibitory concentration; CFU, colony-forming unit; MIC, minimum inhibitory concentration.

Figure 1 shows the relationships between change in log_10_ CFU from baseline of Enterobacterales and each of total-drug plasma and total-drug ELF AUC:MIC ratio based on data from neutropenic murine-thigh and -lung infection models, respectively. Figure 1 shows the relationship between change in log_10_ CFU from baseline of *P. aeruginosa* and total-drug plasma AUC:MIC ratio based on data from a neutropenic murine-thigh infection model. As evidenced by the *r*^2^ values ≥0.84, the data were well described by the fitted functions. A summary of the Hill-type model-based and median AUC:MIC ratio targets associated with efficacy for aminoglycosides against Enterobacterales and *P. aeruginosa* is shown in Table 1.

**Table 1.** Summary of Nonclinical Total-Drug Plasma or ELF AUC:MIC Ratio Targets for Aminoglycoside Efficacy

Organism and Model	Nonclinical Total-Drug Plasma or ELF AUC:MIC Ratio Targets^a,b^					
	Net Bacterial Stasis		1-Log_10_ CFU Reduction from Baseline		2-Log_10_ CFU Reduction from Baseline	
	Hill-Type Model	Median (Min, Max)	Hill-Type Model	Median (Min, Max)	Hill-Type Model	Median (Min, Max)
Enterobacterales^c^						
Thigh	30.7	21.4 (0.592, 98.1)	84.3	62.5 (28.2, 1300)	1030	399 (73.7, 94900)
Lung	14.6	14.5 (11.2, 23.3)	23.7	25.8 (16.4, 36.4)	48.3	75.4 (27.1, 76.6)
*P. aeruginosa* ^d^						
Thigh	58.3	55.4 (36.6, 110)	83.9	74.3 (44.9, 154)	129	128 (51.3, 216)

Abbreviations: AUC, area under the concentration-time curve; AUC:MIC ratio, ratio of the area under the concentration-time curve to minimum inhibitory concentration; CFU, colony-forming unit; ELF, epithelial lining fluid; MIC, minimum inhibitory concentration.

^a^As protein binding for aminoglycosides is low, total-drug plasma AUC:MIC ratio targets were considered.

^b^Based on the assumption that the ratio of the murine total-drug ELF AUC value to total-drug plasma AUC value approaches 1, total-drug plasma AUC:MIC ratio targets for Enterobacterales efficacy based on the neutropenic lung infection model data were referred to as total-drug ELF AUC:MIC ratio targets.

^c^Twelve different Enterobacterales isolates were evaluated in dose-ranging studies carried out using the neutropenic murine-thigh infection model, including 2 *K. pneumoniae* isolates, 4 *E. coli* isolates, 3 *E. cloacae* isolates, and 3 *Serratia marcescens* isolates. Two *K. pneumoniae* isolates were evaluated in the dose-ranging studies carried out using the neutropenic murine-lung infection model.

^d^Five *P. aeruginosa* isolates were evaluated in dose-ranging studies carried out using the neutropenic murine-thigh infection model.

### Simulated Patient Data

The population PK models describing the dispositions of amikacin, gentamicin, and tobramycin in hospitalized patients that were selected from literature [34–36] to use to generate drug exposures in simulated patients are summarized in Supplementary Table 7.

A summary of traditional and extended-interval aminoglycoside dosing regimens by CLcr group that were selected to provide matching total-drug plasma AUC and C_min_ values for simulated patients in each CLcr group compared to those for simulated patients with CLcr >90 to ≤120 mL/min are shown in Table 2. Summary statistics for average total-drug plasma AUC_0–24_ and C_min_ over 48 hours by baseline CLcr group among simulated patients after administration of traditional and extended-interval dosing regimens are shown in Supplementary Tables 8–11. Box-and-whisker plots of these exposure measures among simulated patients by CLcr group after administration of these sets of aminoglycoside dosing regimens are shown in Supplementary Figures 1–6. According to the criteria for achieving drug exposures by CLcr group to match those for normal renal function described in the Supplementary Data, AUC_0–24_ distributions among simulated patients in all CLcr groups were similar to those among simulated patients in the normal renal function group after administration of each of the traditional and extended-interval dosing regimens. The frequency of elevated C_min_ among simulated patients, which was defined as >8 mg/L for amikacin and >2 mg/L for gentamicin or tobramycin, was <20% across all CLcr groups, except for a frequency of 23.8% in the >120 to ≤240 mL/min group for gentamicin and 23.1% in the >30 to ≤45 mL/min group for tobramycin after administration of traditional dosing.

**Table 2.** Summary of Recommended Traditional and Extended-Interval Aminoglycoside Dosing Regimens by CLcr Group^a^

Baseline CLcr, mL/min	Traditional and Extended-Interval Aminoglycoside Dosing Regimens^b^					
	Amikacin		Gentamicin		Tobramycin	
	Traditional Dosing Regimen	Extended-Interval Dosing Regimen	Traditional Dosing Regimen	Extended-Interval Dosing Regimen	Traditional Dosing Regimen	Extended-Interval Dosing Regimen
>120 to ≤240	8 mg/kg q8h	20 mg/kg q24h	2 mg/kg q6h	7 mg/kg q24h	2 mg/kg q6h	7 mg/kg q24h
>90 to ≤120	8 mg/kg q12h	20 mg/kg q24h	2 mg/kg q8h	7 mg/kg q24h	2 mg/kg q8h	7 mg/kg q24h
>60 to ≤90	8 mg/kg q12h	20 mg/kg q36h	1.5 mg/kg q8h	7 mg/kg q36h	1.5 mg/kg q8h	7 mg/kg q36h
>45 to ≤60	8 mg/kg q24h	20mg/kg q48h	1.5 mg/kg q12h	7 mg/kg q48h	1.5 mg/kg q12h	7 mg/kg q48h
>30 to ≤45	8 mg/kg q24h	20 mg/kg q48h	1.5 mg/kg q12h	7 mg/kg q48h	1.5 mg/kg q12h	7 mg/kg q48h
≥16 to ≤30	8 mg/kg q48h	-	2 mg/kg q24h	-	2 mg/kg q24h	-

Abbreviations: ABW, adjusted body weight; AUC, area under the concentration-time curve; CLcr, creatinine clearance; IBW, ideal body weight; TBW, total body weight.

^a^Dosing regimens by CLcr group were selected to provide matching total-drug plasma AUC and C_min_ values for simulated patients in each CLcr group to those for simulated patients with CLcr >90 to ≤120 mL/min. It is important to note that safety for these dosing regimens has not been established.

^b^Dosing regimens were based on TBW. ABW was used in place of TBW if TBW was ≥25% higher than IBW. ABW was calculated for simulated patients as follows: ABW (kg) = IBW + 0.4 • (TBW – IBW) [37, 38].

### Pharmacokinetic–Pharmacodynamic Target Attainment Analyses

Percent probabilities of PK-PD target attainment by MIC based on total-drug plasma and/or ELF AUC:MIC ratio targets associated with net bacterial stasis for Enterobacterales and *P. aeruginosa* based on data from neutropenic murine-thigh and/or -lung infection models among simulated patients with normal renal function after the administration of traditional and extended-interval aminoglycoside dosing regimens are shown in Supplementary Table 12. These results are shown by drug and based on 3 approaches for selecting the AUC:MIC ratio target based on a Hill-type model for a pooled set of isolates, median AUC:MIC ratio target, and randomly assigned AUC:MIC ratio targets. Supplementary Table 13 shows percent probabilities of PK-PD target attainment by MIC based on total-drug plasma and/or ELF AUC:MIC ratio targets associated with a 1-log_10_ CFU reduction from baseline.

Among the set of total-drug plasma AUC:MIC ratio targets derived for the neutropenic murine-thigh infection model for the aminoglycosides against Enterobacterales, there was a high outlying AUC:MIC ratio target associated with the 1-log_10_ CFU reduction from baseline endpoint for 1 isolate. Inclusion of the outlier had a negative impact on results based on randomly assigned targets, while likely having very little impact on the results based on the Hill-type model or median targets. The MIC at which the percent probability of PK-PD target attainment ≥90% was achieved was the same for the Hill-type model and median AUC:MIC ratio targets, and despite the impact of the outlier, it was often the same and almost always within only 1 dilution lower for randomly assigned AUC:MIC ratio targets. Given the similar nature of the findings and some degree of skepticism over the outlying AUC:MIC ratio target value for 1 isolate, percent probabilities of PK-PD target attainment based on the Hill-type model were again (as originally done in 2019) used to support the 2023 USCAST STIC recommendations.

**Figure 2.**
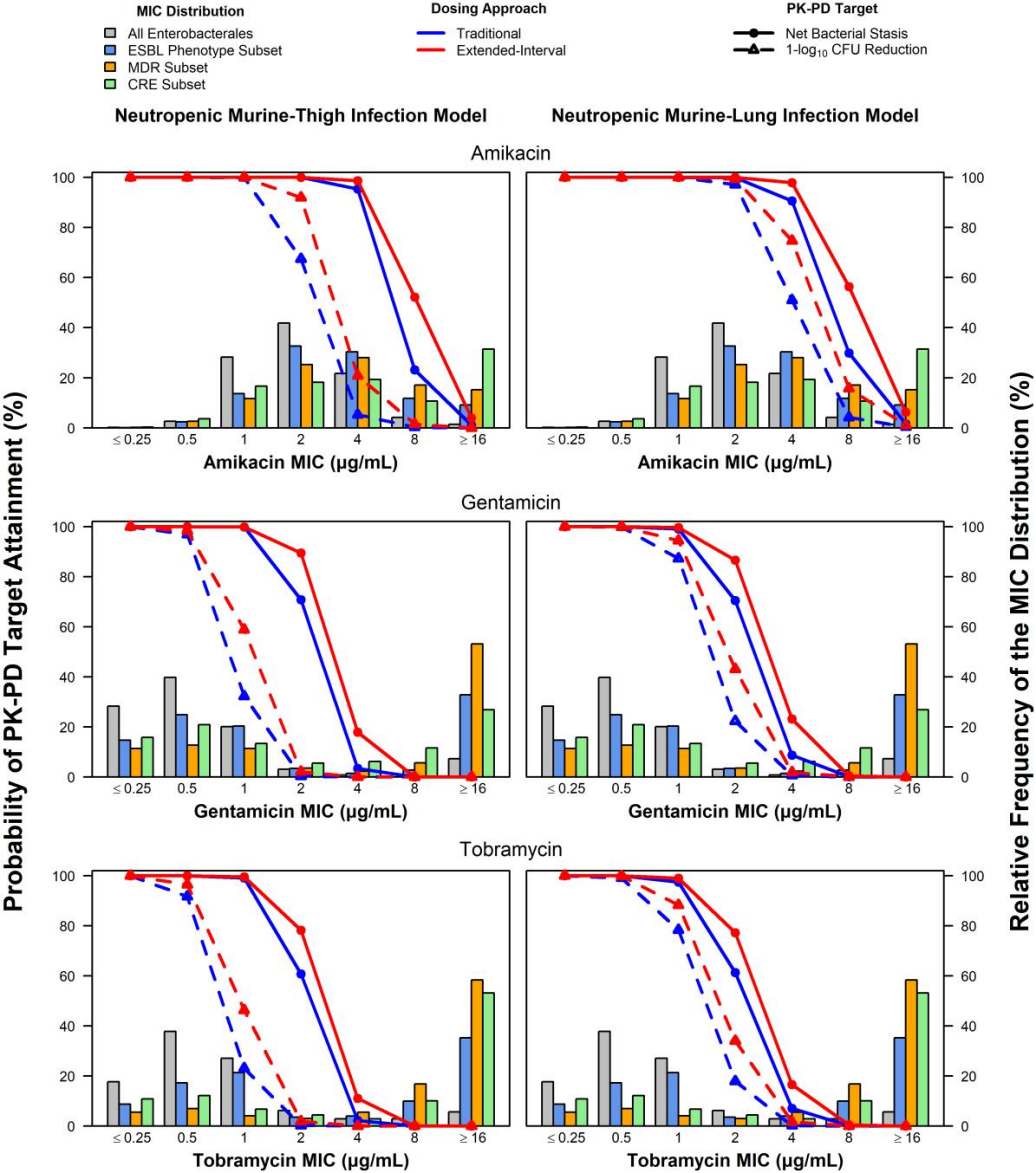
Percent probabilities of PK-PD target attainment by MIC using aminoglycoside total-drug plasma and ELF AUC:MIC ratio targets associated with Enterobacterales efficacy based on pooled data from murine-thigh and -lung infection models, respectively, among simulated patients with normal renal function after administration of traditional and extended-interval dosing regimens. Abbreviations: AUC, area under the concentration-time curve; AUC:MIC ratio, ratio of the area under the concentration-time curve to minimum inhibitory concentration; CRE, carbapenem-resistant Enterobacterales; ELF, epithelial lining fluid; ESBL, extended-spectrum beta-lactamase; MDR, multidrug-resistant; MIC, minimum inhibitory concentration; PK-PD, pharmacokinetic–pharmacodynamic.

**Figure 3.**
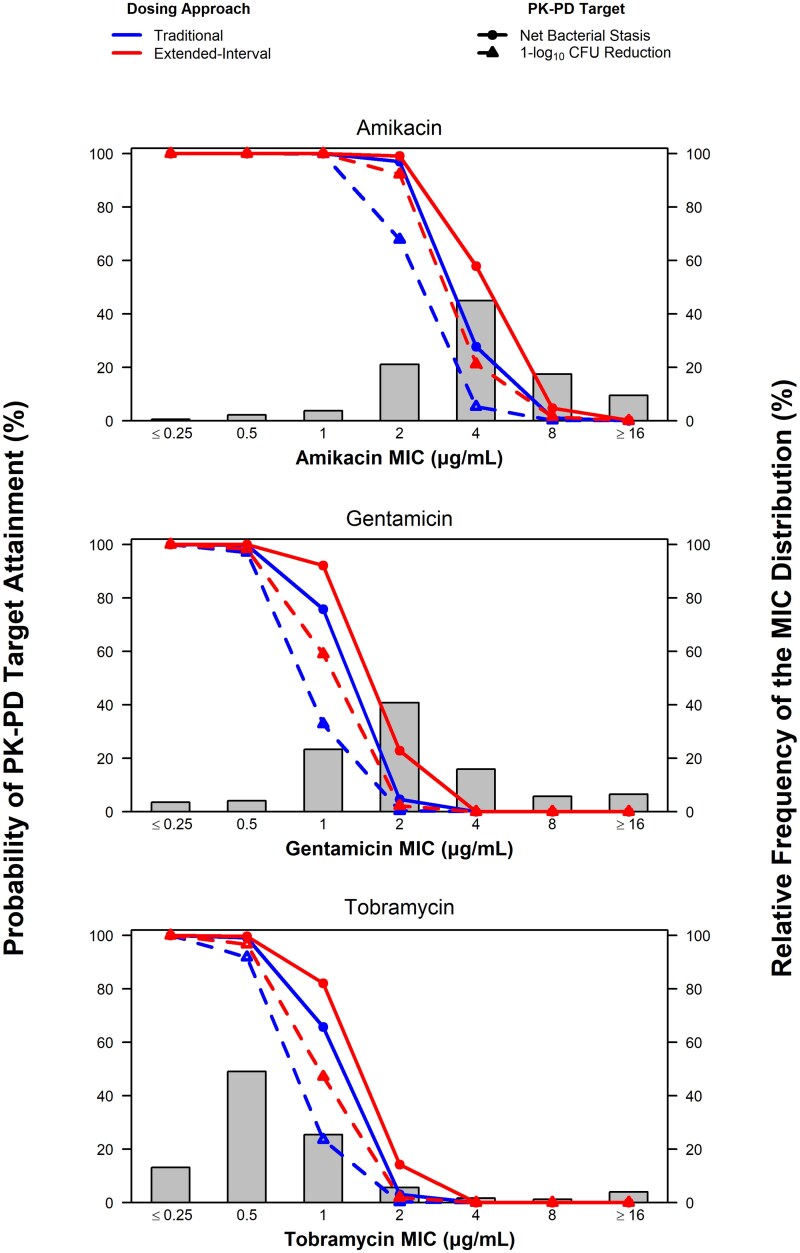
Percent probabilities of PK-PD target attainment by MIC using aminoglycoside total-drug plasma AUC:MIC ratio targets associated with *P. aeruginosa* efficacy based on pooled data from a murine-thigh infection model among simulated patients with normal renal function after administration of traditional and extended-interval dosing regimens. Abbreviations: AUC, area under the concentration-time curve; AUC:MIC ratio, ratio of the area under the concentration-time curve to minimum inhibitory concentration; MIC, minimum inhibitory concentration; PK-PD, pharmacokinetic–pharmacodynamic.

Figures 2 and 3 show the percent probabilities of PK-PD target attainment by MIC based on the Hill-type model AUC:MIC ratio targets for Enterobacterales and *P. aeruginosa* efficacy, respectively, among simulated patients with normal renal function after administration of traditional and extended-interval dosing regimens, overlaid on the MIC distributions for Enterobacterales and the subsets of ESBL phenotype, MDR, and CRE isolates or *P. aeruginosa*.

Percent probabilities of PK-PD target attainment by MIC based on AUC:MIC ratio targets associated with a 1-log_10_ CFU reduction from baseline for Enterobacterales and *P. aeruginosa* derived from the Hill-type models among simulated patients by CLcr group after administration of traditional and extended-interval aminoglycoside dosing regimens are shown in Supplementary Tables 14–19. With the goal of achieving percent probabilities of PK-PD target attainment that approached or were ≥90% based on AUC:MIC ratio targets associated with a 1-log_10_ CFU reduction from baseline derived from the Hill-type models among simulated patients with normal renal function, susceptible breakpoints of 2, 0.5, and 0.5 μg/mL were identified for the amikacin, gentamicin, and tobramycin extended-interval dosing regimens, respectively, against Enterobacterales and *P. aeruginosa*. Corresponding percent probabilities of PK-PD target attainment at these identified MIC values for the remaining CLcr groups are shown in Supplementary Table 20. These results demonstrated that percent probabilities of PK-PD target attainment ≥90% were achieved across all CLcr groups, with only a few exceptions for amikacin in the CLcr >45 to ≤60 and >120 to ≤240 mL/min groups, for which percent probabilities were 79.3% and 69.4%. respectively. These data confirmed that the assessments of the normal renal function group were sufficient as the basis of STIC recommendations for all patients.

### Susceptibility Testing Interpretive Criteria Assessments

Table 3 shows the USCAST-recommended STIC based on the results of the PK-PD target attainment analyses for each aminoglycoside agent against Enterobacterales and *P. aeruginosa*, as well as a comparison of STIC as provided by the CLSI [21], FDA [39], and EUCAST [40]. In the case of the FDA STIC, the CLSI STIC for Enterobacterales and *P. aeruginosa* were recently adopted. For Enterobacterales and *P. aeruginosa*, USCAST-recommended susceptible, S-TDM, and resistant breakpoints were ≤2, 4, and ≥8 µg/mL, respectively, for amikacin and ≤0.5, 1, and ≥2 µg/mL, respectively, for gentamicin and tobramycin. These susceptible breakpoints, which assume the use of extended-interval dosing regimens, represented the highest MIC value at which the percent probabilities of PK-PD target attainment approached or were ≥90% based upon AUC:MIC ratio targets associated with a 1-log_10_ CFU reduction from baseline. The S-TDM breakpoints, which represented the MIC values at which the AUC:MIC ratio associated with a 1-log_10_ CFU reduction from baseline could be achieved using TDM results and dose adjustment, were 1 MIC dilution higher than the susceptible breakpoints. The S-TDM breakpoints for gentamicin or tobramycin of 1 µg/mL represented the highest MIC value at which a total-drug plasma AUC:MIC ratio target of 84 could be achieved while maintaining a total-drug plasma AUC safety threshold of <100 mg•h/L [42, 43]. For amikacin, the S-TDM breakpoint of 4 µg/mL represented the highest such MIC value if the AUC safety threshold were extrapolated up to 400 mg•h/L. As robust evidence demonstrating safety at the exposures necessary for S-TDM is lacking for amikacin, caution is advised.

**Table 3.** USCAST STIC Recommendations for Amikacin, Gentamicin, and Tobramycin Against Enterobacterales and *P. aeruginosa* and Comparisons by Organization

Pathogen/Agent	MIC Breakpoints by Organization, µg/mL			
	USCAST^a^		CLSI^d^/FDA^e^	EUCAST^f^
	2019^b^	2023^c^	Susceptible/Resistant	
	Susceptible/Resistant	Susceptible/Susceptible-TDM/Resistant		
Enterobacterales				
Amikacin	≤4/≥8	≤2/4/≥8	≤4/≥16	≤8/≥16
Gentamicin	≤2/≥4	≤0.5/1/≥2	≤2/≥8	≤2/≥4
Gentamicin-pneumonia	≤1/≥4			
Tobramycin	≤2/≥4	≤0.5/1/≥2	≤2/≥8	≤2/≥4
Tobramycin-pneumonia	≤1/≥4			
*P. aeruginosa*				
Amikacin	≤2/≥8	≤2/4/≥8	≤16/≥64^g^	≤16/≥32
Gentamicin	…	≤0.5/1/≥2	-	-
Tobramycin	≤1/≥2	≤0.5/1/≥2	≤1/≥4	≤2/≥4

Abbreviations: AUC, area under the concentration-time curve; AUC:MIC ratio, ratio of the area under the concentration-time curve to minimum inhibitory concentration; CFU, colony-forming unit; CLSI, Clinical and Laboratory Standards Institute; ECOFF, epidemiological cutoff; ELF, epithelial lining fluid; EUCAST, European Committee on Antimicrobial Susceptibility Testing; FDA, Food and Drug Administration; MIC, minimum inhibitory concentration, PK-PD, pharmacokinetic–pharmacodynamic; S-TDM, susceptible breakpoint based on therapeutic drug monitoring; STIC, susceptibility testing interpretive criteria; TDM, therapeutic drug monitoring; USCAST, United States Committee on Antimicrobial Susceptibility Testing; UTI, urinary tract infection.

^a^Based on the use of the extended-interval aminoglycoside dosing recommendations. Trough concentration monitoring is recommended for safety in all patients receiving aminoglycosides.

^b^Based on the results of PK-PD target attainment analyses evaluating AUC:MIC ratio targets associated with net bacterial stasis and the assumption of combination therapy.

^c^Based on the results of PK-PD target attainment analyses evaluating AUC:MIC ratio targets associated with a 1-log_10_ CFU reduction from baseline. An S-TDM category represents a susceptible breakpoint assuming TDM and dose adjustments to ensure that an AUC:MIC ratio target associated with a 1-log_10_ CFU reduction from baseline of approximately 84 is achieved.

^d^Based on CLSI M100-ED35 (2025) interpretive criteria. STIC are based on population distributions of various species, PK-PD target attainment analyses evaluating AUC:MIC ratio targets associated with net bacterial stasis, and limited clinical data. Combination therapy for most indications other than UTI should be considered [21].

^e^Based on FDA interpretive criteria [39] for which the STIC based on CLSI M100-ED35 [21] is the recognized standard.

^f^Based on EUCAST 2025 breakpoint tables [40]. Represents STIC for systemic infections, which are based on ECOFF values. For such infections, aminoglycosides are recommended to be used in combination with other active therapy [41]. The same STIC are also recommended for infections originating from the urinary tract [40].

^g^Designated as urine STIC, and reporting is only for isolates from the urinary tract [21].

Table 4 shows the implication of different aminoglycoside STIC by organization on percentages of susceptible Enterobacterales and *P. aeruginosa* isolates based on the in vitro surveillance data shown in Supplementary Tables 4 and 5, respectively. Using the USCAST-susceptible breakpoint, the percentages of susceptible Enterobacterales isolates were 72.7%, 68.1%, and 55.4% for amikacin, gentamicin, and tobramycin, respectively. For those institutions that are able to carry out TDM and use such data to adjust dose, up to 94.4%, 88.2%, and 82.5%, respectively, of Enterobacterales isolates would be considered susceptible (ie, using the USCAST S-TDM breakpoint). Importantly, the percentages of susceptible isolates were all lower among resistant subsets, even when considering the S-TDM breakpoint. For *P. aeruginosa*, the percentages of susceptible isolates were 27.9%, 7.6%, and 62.2% for amikacin, gentamicin, and tobramycin, respectively. Using TDM and dose adjustment, the percentages of susceptible isolates were most optimal for tobramycin (87.6%), followed by amikacin (72.9%), but suboptimal for gentamicin (30.9%).

**Table 4.** Implication of Different Aminoglycoside STIC by Organization on Percentages of Susceptible Enterobacterales and *P. aeruginosa* Isolates

Pathogen	Agent	Isolate Collection	MIC Breakpoints by Criteria Organization, µg/mL						
			USCAST^a^			CLSI^b^/FDA^c^		EUCAST^d^	
			STIC: Susceptible/Susceptible-TDM/Resistant	%S	%S-TDM	STIC: Susceptible/Resistant	%S	STIC: Susceptible/Resistant	%S
Enterobacterales	Amikacin	All isolates	≤2/4/≥8	72.7	94.4	≤4/≥16	94.4	≤8/≥16	98.6
		ESBL phenotype subset	…	48.8	79.1	…	79.1	…	90.9
		MDR subset	…	39.7	67.7	…	67.7	…	84.8
		CRE subset	…	38.6	58.0	…	58.0	…	68.6
	Gentamicin	All isolates	≤0.5/1/≥2	68.1	88.2	≤2/≥8	91.2	≤2/≥4	91.2
		ESBL phenotype subset	…	39.5	59.7	…	63.1	…	63.1
		MDR subset	…	24.0	35.4	…	38.9	…	38.9
		CRE subset	…	36.6	50.0	…	55.5	…	55.5
	Tobramycin	All isolates	≤0.5/1/≥2	55.4	82.5	≤2/≥8	88.7	≤2/≥4	88.7
		ESBL phenotype subset	…	26.0	47.3	…	50.8		50.8
		MDR subset	…	12.4	16.4	…	19.4	…	19.4
		CRE subset	…	22.9	29.6	…	34.0	…	34.0
*P. aeruginosa*	Amikacin	All isolates	≤2/4/≥8	27.9	72.9	≤16/≥64^e^	96.2	≤16/≥32	96.2
	Gentamicin	All isolates	≤0.5/1/≥2	7.6	30.9		…	…	…
	Tobramycin	All isolates	≤0.5/1/≥2	62.2	87.6	≤1/≥4	87.6	≤2/≥4	93.2

Abbreviations: AUC, area under the concentration-time curve; AUC:MIC ratio, ratio of the area under the concentration-time curve to minimum inhibitory concentration; CFU, colony-forming unit; CLSI, Clinical and Laboratory Standards Institute; CRE, carbapenem-resistant Enterobacterales; ECOFF, epidemiological cutoff; EUCAST, European Committee on Antimicrobial Susceptibility Testing; MIC, minimum inhibitory concentration, PK-PD, pharmacokinetic–pharmacodynamic; S-TDM, susceptible breakpoint based on therapeutic drug monitoring; STIC, susceptibility testing interpretive criteria; TDM, therapeutic drug monitoring; USCAST, United States Committee on Antimicrobial Susceptibility Testing; UTI, urinary tract infection.

^a^Based on the results of PK-PD target attainment analyses evaluating AUC:MIC ratio targets associated with a 1-log_10_ CFU reduction from baseline and the use of the extended-interval aminoglycoside dosing recommendations. Trough concentration monitoring is recommended for safety in all patients receiving aminoglycosides. An S-TDM category represents a susceptible breakpoint assuming TDM and dose adjustments to ensure that an AUC:MIC ratio target associated with a 1-log_10_ CFU reduction from baseline of approximately 84 is achieved.

^b^Based on CLSI M100-ED35 (2025) interpretive criteria. STIC are based on population distributions of various species, PK-PD target attainment analyses evaluating AUC:MIC ratio targets associated with net bacterial stasis, and limited clinical data. Combination therapy for most indications other than UTI should be considered [21].

^c^Based on FDA interpretive criteria [39] for which the STIC based on CLSI M100-ED35 [21] is the recognized standard.

^d^Based on EUCAST 2025 breakpoint tables [40]. Represents STIC for systemic infections, which are based on ECOFF values. For such infections, aminoglycosides are recommended to be used in combination with other active therapy [41]. The same STIC are also recommended for infections originating from the urinary tract [40].

^e^Designated as urine STIC, and reporting is only for isolates from the urinary tract [21].

## DISCUSSION

The results of the pharmacometric analyses described herein were used to make recommendations for aminoglycoside dosing regimens adjusted for renal function and to re-evaluate the STIC for 3 aminoglycosides, amikacin, gentamicin, and tobramycin, against Enterobacterales and *P. aeruginosa*. The results of the PK-PD target attainment analyses for these agents based on AUC:MIC ratio targets associated with net bacterial stasis were originally interpreted in the context of in vitro surveillance data from 2011 to 2016 collected by the SENTRY Antimicrobial Surveillance Program (JMI Laboratories, North Liberty, IA, USA) and EUCAST data and used to support the USCAST STIC recommendations for Enterobacterales and *P. aeruginosa* made in 2019. The rationale for the use of net bacterial stasis as a nonclinical endpoint was based on the assumption that these agents would be used as part of combination therapy [16]. These results were used to support the re-evaluation of the aminoglycoside STIC carried out by EUCAST [41], followed by the CLSI [21], and recently, the FDA [39]. Like the 2019 USCAST STIC recommendations, the CLSI and FDA also based their STIC recommendations on a net bacterial stasis endpoint [21, 39]. It is important to recognize that until the recent review, susceptible breakpoints in the USA for amikacin, gentamicin, and tobramycin against Enterobacterales and *P. aeruginosa* had been considerably higher for decades, with values ≤16, ≤4, and ≤4, respectively. Thus, while the STIC recommendations were not the same across all 3 groups, the progress made to reassess an older class of antimicrobial agents and establish revised STIC based on contemporary methods represents a major step forward.

Given that USCAST voting members were originally split on whether to base aminoglycoside STIC decisions on AUC:MIC ratio targets associated with net bacterial stasis or a 1-log_10_ CFU reduction from baseline endpoints, the above-described USCAST recommendations were revisited in 2023. It has been suggested that achieving PK-PD targets measured in serum or plasma that are associated with net bacterial stasis may be sufficient for efficacy for infections for which a drug concentrates at the effect site and/or those associated with lower bacterial burdens. For drugs that do not concentrate at the effect site and/or infections associated with higher bacterial burden such as pneumonia, endocarditis, or bacteremia, a 1-log_10_ CFU reduction from baseline may be a more appropriate nonclinical endpoint [44]. In the case of complicated urinary tract infections (cUTI), aminoglycosides concentrate in the urine, and given the drug exposure at the effect site, net bacterial stasis was originally considered an appropriate endpoint for total-drug plasma AUC:MIC ratio targets for Enterobacterales and *P. aeruginosa*. For other indications, this endpoint was considered reasonable as the STIC were based on the assumption of combination therapy. However, for certain types of cUTI (eg, enlarged prostate and foreign body), there is a *locus minoris resistentiae* that represents an infection site with less drug penetration and where the pathogens are slowly growing and, thus, for which it is harder to effect a rapid kill. Given these circumstances and that a 1-log_10_ CFU reduction from baseline endpoint has been the basis for regulatory assessments of dose and STIC for recently developed antimicrobial agents against infections arising from gram-negative infections, including cUTI [45, 46], the 2019 USCAST STIC recommendations for aminoglycosides were revisited. In order to use a consistent metric across new and old agents, the revised USCAST STIC recommendations for aminoglycosides against Enterobacterales and *P. aeruginosa* described herein were based on a 1-log_10_ CFU reduction from baseline endpoint and without a requirement for combination therapy (ie, leaving this decision to the discretion of the clinician). The susceptible breakpoints shown in Table 3 for all 3 agents represented the highest MIC value at which the percent probabilities of PK-PD target attainment associated with this endpoint approached or were ≥90% based on the assessment of extended-interval dosing regimens.

The USCAST STIC re-evaluation of the aminoglycosides also considered that routine TDM for aminoglycosides is carried out in most medical centers in the United States [47]. Although TDM is largely employed in the United States to ensure low trough concentration values to minimize toxicity, these processes can be adapted to calculate patient-specific AUC values, consistent with the recent paradigm shift with vancomycin therapeutic drug monitoring [48]. While the above-described suseptible breakpoints represent the MIC value at which the majority of patients can achieve the AUC:MIC ratio target associated with a 1-log_10_ CFU reduction from baseline with empiric extended-interval dosing, the use of TDM and individualized dose adjustment provide the opportunity to achieve this target in individual patients at an MIC value that is 1 dilution higher. On this basis, USCAST established the S-TDM category for consideration for those institutions for which such implementation of the S-TDM breakpoints could be supported.

The above-described USCAST susceptible and S-TDM breakpoints bisect wild-type MIC distributions, rather than existing high on the upper tails for all 3 aminoglycosides against Enterobacterales and *P. aeruginosa*. Susceptible breakpoints for each agent were 1 to 3 MIC dilutions lower than the 95% ECOFF values and 2 to 5 MIC dilutions lower than the 99.9% ECOFF values shown in Supplementary Table 6. Across the aminoglycoside-pathogen pairs, the percent susceptible based on the recommended susceptible breakpoints ranged from a minimum of 7.6% for gentamicin against *P. aeruginosa* to a maximum of 72.7% for amikacin against Enterobacterales. The S-TDM breakpoints demonstrated better results with ≥82.5%, except for 72.9% and 30.9% for amikacin and gentamicin, respectively, against *P. aeruginosa*. For the MIC distributions of the ESBL phenotype, MDR, and CRE subsets of Enterobacterales, the percent susceptible for the susceptible breakpoint ranged from a minimum of 12.4% for tobramycin with respect to the MDR subset to a maximum of 48.8% for amikacin with respect to the ESBL phenotype subset. These same scenarios determined a range from a minimum of 16.4% to a maximum of 79.1% for the S-TDM breakpoints. The generally low percent susceptible for the ESBL phenotype, MDR, and CRE subsets was also evident relative to the current CLSI and EUCAST susceptible breakpoints, ranging from a minimum of 19.4% for tobramycin against MDR Enterobacterales to a maximum of 90.9% for amikacin against ESBL phenotype subset of Enterobacterales. Thus, aminoglycoside susceptible breakpoints for all organizations demonstrate bisection of the MIC distribution when evaluating the ESBL phenotype, MDR, and CRE subsets of Enterobacterales.

The error in MIC testing at a susceptible breakpoint that bisects an MIC distribution typically impacts a larger percentage of patients than error in MIC testing at the ECOFF value. As a result, many organizations give priority to selecting a susceptible breakpoint such as the ECOFF value that does not bisect an MIC distribution. EUCAST requires that susceptible breakpoints do not bisect the wild-type distribution [24, 49]. However, setting a susceptible breakpoint at a higher MIC value poses a bigger threat to patient care than the error in achieving an MIC result for which the true value is within 1 or more MIC dilutions [50]. In this scenario, the consequence of setting a susceptible breakpoint at the ECOFF value is that a greater percentage of isolates will be categorized as susceptible than would be supported by PK-PD data, thereby inferring greater potency relative to other antimicrobial agents, and potentially putting patients at risk for poor outcomes when treated for “susceptible infections”. Furthermore, a given susceptible breakpoint established at the ECOFF value does not consider the treatment of patients with ESBL phenotype Enterobacterales or CRE, the population for whom treatment guidelines recommend the use of aminoglycosides [32]. As shown for amikacin, the most active of the agents assessed herein, MIC distributions for ESBL phenotype and CRE isolates were shifted to the right, which would also shift the ECOFF values for such subsets. Thus, given the higher prevalence of isolates with elevated MIC values among the patients most likely to receive treatment with aminoglycosides, a susceptible breakpoint, set at the ECOFF value based on the entire collection of isolates, will not achieve the intended purpose given that reproducibility of the MIC test is still a concern among the isolates from resistant subsets in this region of the MIC distribution.

An important output of the pharmacometric analyses undertaken was the traditional and extended-interval aminoglycoside dosing recommendations for patients with renal impairment and augmented renal function (Table 2). The traditional dosing recommendations described in package inserts are vague and limited, stating that dose reductions by either using the normal dose with a prolonged interval or a reduced dose with a fixed interval should be employed [17–19]. Although dosing recommendations to adjust for renal function exist at leading institutions (Supplementary Table 2), they are largely based on nomogram approaches to ensure low C_min_ values and have not been optimized to ensure that AUC values associated with efficacy are achieved. The approach taken to construct these dosing regimens in the present analyses was consistent with contemporary pharmacometric analyses that are undertaken for drugs in development. Such data, which are seldom available for older classes of drugs such as the aminoglycosides, along with the revised STIC recommendations, serve as an important resource for clinicians selecting these agents for treatment. Lastly, it is important to note that extended-interval dose administration allows for drug exposures to be delivered once daily (or less frequently for patients with renal impairment), which, relative to traditional-interval dose administration, optimizes efficacy given the concentration-dependent pattern of killing observed for this class [25] and safety by allowing for lower C_min_ values to be achieved. Thus, the traditional dosing regimens adjusted for renal function have less utility given this paradigm but are provided herein as a resource to clinicians.

STIC evaluations are ideally informed from 3 data sources. These include in vitro surveillance data to evaluate MIC distributions, clinical response by MIC data from patients, and pharmacometric analyses that include the use of nonclinical PK-PD targets for efficacy, population PK models, and simulation to carry out PK-PD target attainment analyses. As described previously [16] and based on the CLSI's recent review [51], clinical outcome by MIC data to support STIC recommendations for aminoglycosides were limited. This limitation is to be expected for STIC evaluations of older classes of agents. Another limitation of these analyses was that in vivo data for efficacy could have been more robust. Dose-ranging efficacy data for aminoglycosides against *P. aeruginosa* from a neutropenic murine-lung infection model were not available. Furthermore, direct aminoglycoside total-drug ELF AUC:MIC ratio targets for efficacy were inferred using external murine ELF penetration ratio data [52, 53] as murine ELF PK were not originally characterized.

In summary, the revised USCAST STIC for amikacin, gentamicin, and tobramycin against Enterobacterales and *P. aeruginosa* were based on a critical examination of the pharmacometric-based analysis results using extended-interval dosing regimens, which optimize drug exposure at infection sites and minimize the risk of toxicity [13, 14]. Use of aminoglycoside dosing recommendations for patients with renal impairment and augmented renal function and USCAST-recommended susceptible breakpoints for use with dose adjustments using TDM, as described herein, will further the ability of clinicians to safely and effectively utilize this class of agents.

## Supplementary Material

ofaf426_Supplementary_Data

## Supplementary Data

Supplementary materials are available at *Open Forum Infectious Diseases* online. Consisting of data provided by the authors to benefit the reader, the posted materials are not copyedited and are the sole responsibility of the authors, so questions or comments should be addressed to the corresponding author.
