# Supplementary material for: Evaluation of Aminoglycoside Dosing Regimens Adjusted for Renal Function and In Vitro Susceptibility Test Interpretive Criteria for Enterobacterales and Pseudomonas aeruginosa
Source: Open Forum Infect Dis. 2025 Nov 26;12(12):ofaf426. doi: 10.1093/ofid/ofaf426 (PMC12651555; doi:10.1093/ofid/ofaf426)
Supplement: ofaf426_Supplementary_Data [file ofaf426_supplementary_data.pdf]

SUPPLEMENTAL MATERIAL

Supplemental Methods

Non-Clinical Pharmacokinetic-Pharmacodynamic Studies

Animals were maintained in accordance with the guidelines of the Association for Assessment and Accreditation of Laboratory Animal Care International. All animal studies were approved by the Animal Research Committees of the William S. Middleton Memorial VA Hospital and the University of Wisconsin.

For the dose-ranging studies carried out for amikacin, gentamicin, and tobramycin against Enterobacterales and *P. aeruginosa* using neutropenic murine-thigh and -lung infection models, mice were rendered neutropenic by cyclophosphamide administered by intra-peritoneal injection four days (150 mg/kg of body weight) and one day (100 mg/kg) prior to experimental infection. In all studies, treatment was initiated 2 hours after inoculation of $10^{5\text{-}6}$ and $10^{7\text{-}8}$ CFU of the challenge isolate into the posterior thighs or the lungs of mice. All studies included untreated control arms. Treated mice were sacrificed at the end of 24 hours of therapy, while groups of control mice were sacrificed just prior to the time treated mice were administered the first dose of antibiotic and after 24 hours.

The overarching design of the dose-ranging studies was to evaluate amikacin, gentamicin, and tobramycin activity in mice infected with 12 different Enterobacterales isolates, including *E. coli*, *E. cloacae*, *K. pneumoniae*, and *S. marcescens* isolates, and also *P. aeruginosa* isolates. The challenge isolate panels included bacteria with varying aminoglycoside MIC values (0.125 to 16 µg/mL) exposed to doses ranging from 0.125 to 1536 mg/kg, administered using a variety of dosing intervals (every 2, 3, 4, 6, 8, and 12

hours) with adequate dose range and dosing intervals tailored individually to each isolate-drug pairing.

Using the data from these studies, the relationship between log_{10} CFU reduction from baseline at 24 hours and total-drug plasma AUC:MIC ratio were evaluated using Hill-type models and nonlinear least-squares regression by pathogen or pathogen group. The magnitude of the total-drug plasma AUC:MIC ratio associated with net bacterial stasis and 1- and 2-log_{10} CFU reductions from baseline for Enterobacterales and *P. aeruginosa* were determined. The binding of aminoglycosides to plasma proteins, including in mice, is low. Thus, no corrections were made for total-drug plasma AUC:MIC ratio targets by agent and this was the basis for pooling data for three agents when evaluating the above-described PK-PD relationships for efficacy.

AUC:MIC ratio targets for efficacy based on murine ELF exposure were not determined as a part of the above-described studies using the neutropenic murine-lung infection model. However, previous studies have demonstrated the penetration of aminoglycosides into murine ELF, as determined by the ratio of the ELF AUC value to total-drug plasma AUC values, to range from 0.8 to 1.0 [1, 2]. Given this range for ELF penetration, total-drug plasma AUC:MIC ratio targets determined based on data from the neutropenic murine-lung infection model were assumed to be equivalent in magnitude to those based on ELF exposures. In the context of this assumption, total-drug plasma AUC:MIC ratio targets for Enterobacterales efficacy based on the neutropenic murine-lung infection model data were herein referred to as total-drug ELF AUC:MIC ratio targets.

Simulated Patient Data

Generation of Aminoglycoside Concentration-Time Profiles for Simulated Patients

Plasma concentration-time profiles were generated for each simulated patient after administration of the traditional and extended-interval dosing regimens for each agent described below using an appropriate population PK model, implemented in R version 3.3.1 [3] using the mrgsolve package [4]. Population PK models for each of amikacin, gentamicin, and tobramycin from the published literature were selected to carry out simulations. Preference was given to those population PK models constructed using data from the target patient population(s). The criteria used to discriminate among candidate population PK models and the process to generate individual PK parameter values for each agent among simulated patients using the final model selected are described below.

The criteria used to discriminate among candidate population PK models identified from the literature included the following: (1) Evaluation of individual and population mean parameter estimates and their precision (e.g., standard error of the mean); (2) Graphical examination of standard goodness-of-fit diagnostic plots and the observed versus individual predicted drug concentration-time profiles, as a measure of precision; and (3) Evaluation of interindividual and residual variability, patient population, and reproducibility. Models developed in adults ≥ 18 years, in which relevant covariates were assessed (renal function for clearance; body size for volumes), in which the structural and statistical model was reproducible from the original publication, and for which the patient population was generalizable to patients with a broad set of indications were prioritized.

Criteria to Select Aminoglycoside Dosing Regimens by CLcr Group

Traditional or extended-interval dosing regimens for each agent identified for a given CLcr group were selected to provide matching total-drug plasma AUC and C_{min} values for dosing regimens administered to simulated patients with normal renal function (i.e., CLcr > 90 to ≤ 120 mL/min). Dosing regimens by CLcr group were selected using the following criteria: (1) ≤ 50% difference in median of the AUC from time 0 to 24 hours (AUC_{0-24}) values among simulated patients in each baseline CLcr group as compared to those with CLcr > 90 to ≤ 120 mL/min; (2) ≤ 25% of simulated AUC_{0-24} values contained in the tails (5th, 95th percentiles) of the AUC_{0-24} distribution for simulated patients with CLcr > 90 to ≤ 120 mL/min; (3) ≤ 20% of simulated patients with C_{min} values > 8 mg/L for amikacin and > 2 mg/L for gentamicin or tobramycin in each CLcr group [5, 6, 7, 8].

Aminoglycoside Protein Binding and ELF Penetration Ratio

The range of protein binding point estimates for each aminoglycoside agent obtained from the package inserts [6, 7, 8] are summarized in **Table S3**. The binding of aminoglycosides to plasma or serum proteins is low and is dependent upon test conditions, especially the concentrations of the divalent cations, calcium, and magnesium [9, 10, 11, 12, 13]. Many determinations of protein binding in the presence of divalent cations approximate zero. Thus, it was considered reasonable to assume negligible protein binding and base PK-PD target attainment analyses on total-drug plasma rather than free-drug plasma AUC values.

Average total-drug ELF AUC_{0-24} values over 48 hours were generated for simulated patients by multiplying total-drug plasma AUC_{0-24} values by an ELF penetration ratio. A penetration ratio was randomly assigned to each simulated patient based on a distribution of ratios from a previous PK analysis of tobramycin, which was carried out to quantify ELF penetration in patients with pneumonia [14, 15]. Although data describing the ELF penetration ratio was only available for tobramycin, the extent of ELF penetration is assumed to be similar for gentamicin and amikacin.

Supplemental Results

Susceptibility Testing Interpretive Criteria Assessments

The USCAST 2019 recommendations removed the gentamicin STIC for *P. aeruginosa*. The new recommendations have reinstated these STIC in an effort to demonstrate to clinicians the low susceptibility of gentamicin against *P. aeruginosa*. As seen in **Table 4,** only 7.6% of all *P. aeruginosa* isolates would be considered susceptible to gentamicin. Up to 30.9% of all isolates would be considered susceptible using TDM. It was the belief of USCAST that the best approach for showing that gentamicin is not an effective agent for *P. aeruginosa* infections was to establish appropriate STIC for this pair, and subsequently having most isolates test as non-susceptible for clinicians to see this result when managing patients. Given that so few isolates test as susceptible to gentamicin with existing STIC and that many institutions have already removed gentamicin from their panel, it is reasonable to continue to not include gentamicin on the susceptibility panels for *P. aeruginosa* as most isolates are resistant.

Supplemental Tables

Table S1. Summary of aminoglycoside dosing regimens based on baseline renal function described in FDA package inserts

Baseline CLcr (mL/min)	Aminoglycoside dosing regimen		
	Amikacin[a,b]	Gentamicin[c,d]	Tobramycin[e,f]
> 90 to ≤ 120[g]	7.5 mg/kg IV q12h or 5 mg/kg IV q8h	1 to 1.7 mg/kg IV q8h	1 to 1.7 mg/kg IV q8h
> 60 to ≤ 90	Normal dose + prolonged interval or reduced dose + fixed interval	Normal dose + prolonged interval or reduced dose + fixed interval	Normal dose + prolonged interval or reduced dose + fixed interval
> 50 to ≤ 60			
> 40 to ≤ 50			
> 30 to ≤ 40			
≥ 16 to ≤ 30			

a. If the CLcr is not available and the patient was stable, the dosing interval, in hours, was calculated by multiplying the Scr by 9 [6].
b. If serum assay was not available and patient was stable, therapy was initiated with 7.5 mg/kg as a loading dose. The maintenance dose administered q12h was calculated using a formula [6].
c. The interval between doses, in hours, was calculated by multiplying the Scr by 8 [7].
d. After the usual initial dose, the amount of the reduced dose administered every 8 hours was calculated by multiplying the normal dose by the percent of the normal dose from the nomogram [7].
e. If the CLcr was not available and the patient was stable, the dosing interval, in hours, was calculated by multiplying the Scr by 6 [8].
f. When the CLcr was ≤ 70 mL/min, the amount reduced dose was calculated by multiplying the normal dose by the percent of the normal dose from the nomogram [8].
g. While normal renal function was not defined by a given CLcr range, this was considered to be CLcr > 90 to ≤ 120 mL/min.

Table S2. Summary of traditional and extended-interval aminoglycoside dosing regimens based on baseline renal function as described in dosing recommendations from selected USA hospitals[a]

Baseline CLcr, (mL/min)	Traditional and extended-interval aminoglycoside dosing regimens					
	Amikacin		Gentamicin		Tobramycin	
	Traditional	Extended-interval	Traditional	Extended-interval	Traditional	Extended-interval
> 90 to ≤ 120	5-8 mg/kg q12h	15-20 mg/kg q24h	1.5-2 mg/kg q8h	5-7 mg/kg q24h	1.5-2 mg/kg q8h	5-7 mg/kg q24h
> 60 to ≤ 90	5-8 mg/kg q12h	15-20 mg/kg q24h	1.5-2 mg/kg q8h	5-7 mg/kg q24h	1.5-2 mg/kg q8h	5-7 mg/kg q24h
> 50 to ≤ 60	5-8 mg/kg q12h	15-20 mg/kg q36h	1.5-2 mg/kg q12h	5-7 mg/kg q36h	1.5-2 mg/kg q12h	5-7 mg/kg q36h
> 40 to ≤ 50	5-8 mg/kg q12h	15-20 mg/kg q36h	1.5-2 mg/kg q12h	5-7 mg/kg q36h	1.5-2 mg/kg q12h	5-7 mg/kg q36h
> 30 to ≤ 40	5-8 mg/kg q24h	15-20 mg/kg q48h	1.5-2 mg/kg q24h	5-7 mg/kg q48h	1.5-2 mg/kg q24h	5-7 mg/kg q48h
≥ 16 to ≤ 30	5-8 mg/kg q48h	Contraindicated	1.5-2 mg/kg q48h	Contraindicated	1.5-2 mg/kg q48h	Contraindicated

a. Based on dosing recommendations from nine USA hospitals deemed to have best practices [16].

Table S3. Protein binding estimates of amikacin, gentamicin, and tobramycin

Agent	Protein binding estimate
Amikacin	0-11% [6]
Gentamicin	0-30% [7]
Tobramycin	0% [8]

Table S4. Aminoglycoside MIC distributions for Enterobacterales isolates[a] and subsets of resistant isolates collected from medical centers in the USA from 2017 to 2021 as part of the Sentry Antimicrobial Surveillance Program

Isolate collection	Agent (n)	Number of isolates (cumulative % inhibited) by MIC (µg/mL)[b]										$MIC_{50/90}$ (µg/mL)	
		0.12	0.25	0.5	1	2	4	8	16	32	>	MIC_{50}	MIC_{90}
All Enterobacterales	Amikacin<br>(n=47,130)		71<br>(0.2)	1,213<br>(2.7)	13,284<br>(30.9)	19,709<br>(72.7)	10,223<br>(94.4)	1,953<br>(98.6)	394<br>(99.4)	174<br>(99.8)	109<br>(100)	2	4
	Gentamicin<br>(47,123)	908<br>(1.9)	12,422<br>(28.3)	18,750<br>(68.1)	9,477<br>(88.2)	1,420<br>(91.2)	355<br>(92.0)	389<br>(92.8)	336<br>(93.5)		3,066<br>(100)	0.5	2
	Tobramycin<br>(n=47,129)	333<br>(0.7)	7,993<br>(17.7)	17,790<br>(55.4)	12,756<br>(82.5)	2,923<br>(88.7)	1,353<br>(91.6)	1,335<br>(94.4)	1,194<br>(96.9)		1,452<br>(100.0)	0.5	4
ESBL phenotype	Amikacin<br>(n=5,138)		5<br>(0.1)	122<br>(2.5)	701<br>(16.1)	1,680<br>(48.8)	1,557<br>(79.1)	603<br>(90.9)	245<br>(95.6)	134<br>(98.2)	91<br>(100)	4	8
	Gentamicin<br>(n=5,137)	79<br>(1.5)	675<br>(14.7)	1,274<br>(39.5)	1,041<br>(59.7)	173<br>(63.1)	72<br>(64.5)	141<br>(67.3)	115<br>(69.5)		1,567<br>(100)	1	>16
	Tobramycin<br>(n=5,138)	30<br>(0.6)	420<br>(8.8)	884<br>(26.0)	1,094<br>(47.3)	183<br>(50.8)	206<br>(54.8)	510<br>(64.8)	732<br>(79.0)		1,079<br>(100)	2	>16
MDR subset	Amikacin<br>(n=3,612)		6<br>(0.2)	92<br>(2.7)	423<br>(14.4)	914<br>(39.7)	1,012<br>(67.7)	616<br>(84.8)	290<br>(92.8)	159<br>(97.2)	100<br>(100.0)	4	16
	Gentamicin<br>(n=3,611)	53<br>(1.5)	356<br>(11.3)	458<br>(24.0)	411<br>(35.4)	126<br>(38.9)	84<br>(41.2)	204<br>(46.9)	154<br>(51.1)		1,765<br>(100)	16	>16
	Tobramycin<br>(n=3,612)	21<br>(0.6)	178<br>(5.5)	249<br>(12.4)	145<br>(16.4)	106<br>(19.4)	200<br>(24.9)	607<br>(41.7)	866<br>(65.7)		1,240<br>(100)	16	>16
CRE subset	Amikacin<br>(n=621)		2<br>(0.3)	22<br>(3.9)	103<br>(20.5)	113<br>(38.6)	120<br>(58.0)	66<br>(68.6)	59<br>(78.1)	75<br>(90.2)	61<br>(100)	4	32
	Gentamicin<br>(n=620)	16<br>(2.6)	82<br>(15.8)	129<br>(36.6)	83<br>(50.0)	34<br>(55.5)	38<br>(61.6)	72<br>(73.2)	28<br>(77.7)		138<br>(100)	1	>16
	Tobramycin<br>(n=621)	8<br>(1.3)	59<br>(10.8)	75<br>(22.9)	42<br>(29.6)	27<br>(34.0)	18<br>(36.9)	62<br>(46.9)	117<br>(65.7)		213<br>(100)	16	>16

a. Isolates were collected from 81 medical centers across 36 states from all 9 US Census Divisions.
b. Shaded cells represent the MIC values up to and/or including the MIC_{90} value.

Table S5. Aminoglycoside MIC distributions for *P. aeruginosa* isolates[a] collected from medical centers in the USA from 2017 to 2021 as part of the Sentry Antimicrobial Surveillance Program

Agent (n)	Number of isolates (cumulative % inhibited) by MIC (µg/mL)[b]													$MIC_{50/90}$ (µg/mL)	
	0.06	0.12	0.25	0.5	1	2	4	8	16	32	64	128	>	MIC_{50}	MIC_{90}
Amikacin (n=8,906)			55 (0.6)	205 (2.9)	335 (6.7)	1,888 (27.9)	4,012 (72.9)	1,561 (90.5)	514 (96.2)	170 (98.1)			166 (100)	4	8
Gentamicin (n=8,904)		182 (2.0)	134 (3.5)	364 (7.6)	2,073 (30.9)	3,627 (71.7)	1,421 (87.6)	517 (93.4)	168 (95.3)				418 (100)	2	8
Tobramycin (n=8,905)		253 (2.8)	917 (13.1)	4,369 (62.2)	2,259 (87.6)	502 (93.2)	149 (94.9)	103 (96.0)	61 (96.7)				292 (100)	0.5	2

a. Isolates were collected from 81 medical centers across 36 states from all 9 US Census Divisions.
b. Shaded cells represent the MIC values up to and/or including the MIC_{90} value.

Table S6. Aminoglycoside ECOFF values for Enterobacterales and *P. aeruginosa* isolates[a] collected from medical centers in the USA from 2017 to 2021 as part of the Sentry Antimicrobial Surveillance Program

Pathogen	Agent	ECOFF in µg/mL based on SENTRY data[b]				
		**95.0%**	**97.5%**	**99.0%**	**99.5%**	**99.9%**
Enterobacterales	Amikacin	4	8	8	8	16
	Gentamicin	1	2	2	2	2
	Tobramycin	2	2	2	2	4
P. aeruginosa	Amikacin	8	16	16	16	32
	Gentamicin	4	8	8	8	16
	Tobramycin	1	2	2	2	2

a. Based on all isolates collected from 81 medical centers across 36 states from all 9 US Census Divisions.
b. Note, ECOFF values were calculated as described by EUCAST [17, 18]

Table S7. Selected population pharmacokinetic models

Agent	Author	Structure	Population	Parameter means[a]	Covariates[b]		Interindividual variability (IIV)[c]		Residual variability (RV)[d]
Amikacin	Romano *et al.* [19]	one-compartment	158 adults admitted to the ICU	CL – 4.48<br>VC – 15.6	CLcr on CL	WT on VC	CL – 28.2%	VC – 23.2%	22%
Gentamicin	Xuan *et al.* [20]	two-compartment	939 adult inpatients	CL – 4.32<br>VC – 19.7<br>k12 – 0.092<br>k21 – 0.071	CLcr on CL	WT on VC	CL – 29.6%<br>k12 – 69.9%<br>k21 – 63.9%	VC – 5.83%	23.7%<br>0.23 mg/L
Tobramycin	Aarons *et al.* [21]	two-compartment	97 adult inpatients	CL – 4.01<br>VC – 21.7<br>k12 – 0.012<br>k21 – 0.027	CLcr on CL	WT on VC	CL – 32%	VC – 3%	21%

a. CL – total clearance (L/h); VC – central volume of distribution (L); k12 – rate constant for transfer from central to peripheral compartment (h^{-1}); k21 – rate constant for transfer from peripheral to central compartment (h^{-1}).
b. CLcr – Cockcroft-Gault creatinine clearance in mL/min; WT – weight in kg.
c. Interindividual variability, expressed as CV% from a log-normal distribution.
d. Residual variability (RV), expressed as CV% for proportional terms and in mg/L for additive terms.

Table S8. Summary statistics for average total-drug plasma AUC_{0-24} over 48 hours among simulated patients by CLcr group after administration of traditional aminoglycoside dosing regimens

Agent	Baseline CLcr group (mL/min)	Comparisons relative to > 90 to ≤ 120			Summary statistics for average total-drug plasma AUC_{0-24} (mg•h/L)								
		< 5th% tile	≥ 5th% to ≤ 95th% tile	> 95th% tile	Mean	%CV	Min	5th %tile	25th %tile	Median	75th %tile	95th %tile	Max
Amikacin	>120 to ≤240	2.3	84.7	13.0	247.18	39.12	77.85	134.43	185.48	226.68	287.31	422.00	1032.52
	>90 to ≤120	5.0	90.0	5.0	208.95	39.76	82.37	123.99	155.96	193.56	241.27	338.81	1254.78
	>60 to ≤90	1.0	84.7	14.3	260.10	33.66	95.10	152.94	201.75	243.84	298.69	424.98	967.13
	>45 to ≤60	16.1	81.7	2.2	181.07	36.55	62.54	100.07	137.88	168.85	213.29	301.36	674.33
	>30 to ≤45	2.7	91.6	5.7	220.51	29.98	72.58	134.74	175.31	210.75	251.80	344.20	553.73
	≥16 to ≤30	11.9	87.8	0.3	175.75	26.53	66.96	109.97	145.26	170.30	202.25	258.80	417.85
Gentamicin	>120 to ≤240	3.6	85.2	11.2	83.08	30.94	23.98	46.96	64.02	80.04	98.75	129.55	185.81
	>90 to ≤120	5.0	90.0	5.0	75.79	30.19	27.09	44.83	58.93	73.22	89.16	114.78	199.16
	>60 to ≤90	9.7	88.2	2.1	68.84	29.21	22.26	39.66	54.88	66.83	81.25	104.37	170.15
	>45 to ≤60	23.1	76.1	0.8	58.72	30.68	16.93	31.80	45.88	57.80	69.79	89.84	136.77
	>30 to ≤45	11.0	87.2	1.8	67.75	28.94	18.54	38.38	53.67	65.68	79.69	102.31	136.04
	≥16 to ≤30	16.5	83.2	0.3	64.31	29.97	13.83	34.13	50.56	64.06	77.55	97.26	121.85
Tobramycin	>120 to ≤240	4.2	87.5	8.3	74.52	33.49	22.85	39.64	56.54	71.31	88.47	118.17	188.07
	>90 to ≤120	5.0	90.0	5.0	69.58	32.58	22.61	38.08	53.16	66.63	82.08	110.49	188.87
	>60 to ≤90	6.7	90.4	2.9	64.95	31.69	20.80	36.70	49.58	62.78	77.37	102.10	151.17
	>45 to ≤60	11.6	88.1	0.3	57.75	29.53	17.80	32.36	45.65	56.28	68.38	88.76	121.72
	>30 to ≤45	3.1	95.6	1.3	69.73	25.02	24.40	41.55	57.96	68.37	81.11	98.94	130.59
	≥16 to ≤30	1.6	97.6	0.8	70.18	23.59	25.24	43.43	57.90	70.37	81.80	97.05	128.61

Table S9. Summary statistics for average total-drug plasma C_{min} over 48 hours by baseline CLcr group among simulated patients after administration of traditional aminoglycoside dosing regimens

Agent	Baseline CLcr group (mL/min)	Comparisons relative to > 90 to ≤ 120			% above threshold[a]	Summary statistics for average total-drug plasma C_{min} (mg/L)								
		< 5th% tile	≥ 5th% to ≤ 95th% tile	> 95th% tile		Mean	%CV	Min	5th %tile	25th %tile	Median	75th %tile	95th %tile	Max
Amikacin	>120 to ≤240	2.5	84.8	12.7	10.8	3.99	90.20	0.01	0.46	1.67	3.06	5.17	10.58	36.00
	>90 to ≤120	5.0	90.0	5.0	4.3	2.93	99.50	0	0.30	1.13	2.23	3.81	7.45	40.11
	>60 to ≤90	1.6	81.9	16.5	13.6	4.62	70.80	0.05	0.82	2.30	3.84	6.40	10.72	29.45
	>45 to ≤60	12	85.6	2.4	1.7	2.10	93.24	0	0.10	0.70	1.64	2.87	5.74	17.18
	>30 to ≤45	3.0	93.2	3.8	2.5	3.24	62.66	0	0.54	1.77	2.94	4.26	6.97	13.53
	≥16 to ≤30	6.9	91.1	2.0	1.4	2.50	75.18	0	0.23	1.06	2.23	3.44	5.95	14.29
Gentamicin	>120 to ≤240	4.8	84.0	11.2	23.8	1.48	59.73	0.04	0.38	0.84	1.30	1.96	3.09	5.62
	>90 to ≤120	5.0	90.0	5.0	16.3	1.31	57.81	0.08	0.37	0.75	1.16	1.73	2.63	5.62
	>60 to ≤90	3.3	90.3	6.4	18.8	1.41	50.17	0.10	0.47	0.89	1.29	1.79	2.74	4.91
	>45 to ≤60	6.5	92.0	1.5	7.6	1.11	52.26	0.04	0.32	0.65	1.03	1.46	2.18	3.72
	>30 to ≤45	1.8	93.5	4.7	18.0	1.44	44.52	0.08	0.53	0.97	1.37	1.83	2.60	3.68
	≥16 to ≤30	4.3	95.6	0.1	7.2	1.15	44.75	0.13	0.39	0.75	1.10	1.48	2.11	2.64
Tobramycin	>120 to ≤240	3.9	88.0	8.1	18.4	1.27	69.11	0.02	0.21	0.62	1.10	1.70	2.91	5.78
	>90 to ≤120	5.0	90.0	5.0	13.3	1.13	68.69	0.02	0.19	0.55	0.99	1.54	2.54	5.57
	>60 to ≤90	2.5	89.8	7.7	18.0	1.31	59.04	0.05	0.30	0.71	1.19	1.78	2.75	4.49
	>45 to ≤60	3.5	94.2	2.3	8.2	1.06	58.01	0.02	0.22	0.60	0.95	1.43	2.25	3.36
	>30 to ≤45	1.0	92.1	6.9	23.1	1.51	43.59	0.07	0.44	1.05	1.47	1.94	2.68	3.71
	≥16 to ≤30	1.3	98.1	0.6	7.1	1.19	46.27	0.03	0.33	0.77	1.18	1.60	2.11	3.01

a. Safety thresholds assessed for total-drug plasma C_{min} were ≥ 8 mg/L for amikacin and ≥ 2 mg/L for gentamicin and tobramycin [5, 6, 7, 8].

Table S10. Summary statistics for average total-drug plasma AUC_{0-24} over 48 hours among simulated patients by CLcr group after administration of extended-interval aminoglycoside dosing regimens

Agent	Baseline CLcr group (mL/min)	Comparisons relative to > 90 to ≤ 120			Summary statistics for average total-drug plasma AUC_{0-24} (mg•h/L)								
		< 5th% tile	≥ 5th% to ≤ 95th% tile	> 95th% tile	Mean	%CV	Min	5th %tile	25th %tile	Median	75th %tile	95th %tile	Max
Amikacin	>120 to ≤240	21.0	76.9	2.1	217.22	39.84	71.13	116.68	159.99	200.99	250.88	381.92	714.39
	>90 to ≤120	5.0	90.0	5.0	275.30	41.79	88.55	153.00	201.52	249.99	316.02	462.41	1254.79
	>60 to ≤90	0.3	91.9	7.8	310.58	30.55	138.74	189.63	245.61	292.48	355.25	493.34	861.97
	>45 to ≤60	13.9	83.8	2.3	238.06	39.89	88.99	126.58	177.11	223.32	277.52	402.91	1099.38
	>30 to ≤45	1.6	91.3	7.1	307.78	33.53	116.77	184.49	240.95	288.00	355.85	488.00	869.30
	≥16 to ≤30	-	-	-	-	-	-	-	-	-	-	-	-
Gentamicin	>120 to ≤240	16.4	82.2	1.40	78.23	33.02	24.96	40.82	59.23	76.12	92.99	123.87	196.89
	>90 to ≤120	5.0	90.0	5.0	95.57	32.34	29.41	53.17	74.15	91.09	113.22	151.81	217.73
	>60 to ≤90	1.2	89.6	9.2	105.79	28.34	44.13	62.72	83.99	101.15	124.15	161.42	239.27
	>45 to ≤60	15.3	83.4	1.3	79.91	33.65	30.09	43.12	60.44	76.32	95.24	130.36	201.99
	>30 to ≤45	4.2	90.9	4.9	98.53	30.28	24.10	55.25	78.16	95.15	115.98	151.21	244.16
	≥16 to ≤30	-	-	-	-	-	-	-	-	-	-	-	-
Tobramycin	>120 to ≤240	14.8	84.3	0.9	67.00	35.24	14.17	33.41	50.75	63.46	80.22	110.31	164.16
	>90 to ≤120	5.0	90.0	5.0	85.69	35.62	27.43	43.80	64.17	81.32	103.16	137.97	219.05
	>60 to ≤90	1.0	88.0	11.0	99.65	29.90	33.90	56.30	77.92	96.87	118.17	153.97	205.57
	>45 to ≤60	9.1	88.5	2.4	73.80	35.51	19.51	39.33	54.60	69.99	87.17	123.60	190.27
	>30 to ≤45	1.9	90.4	7.7	94.87	31.07	32.49	50.13	73.32	90.88	113.03	146.16	222.27
	≥16 to ≤30	-	-	-	-	-	-	-	-	-	-	-	-

Table S11. Summary statistics for average total-drug plasma C_{min} over 48 hours among simulated patients by CLcr group after administration of extended-interval aminoglycoside dosing regimens

Agent	Baseline CLcr group (mL/min)	Comparisons relative to > 90 to ≤ 120			% above threshold[a]	Summary statistics for average total-drug plasma C_{min} (mg/L)								
		< 5th% tile	≥ 5th% to ≤ 95th% tile	> 95th% tile		Mean	%CV	Min	5th %tile	25th %tile	Median	75th %tile	95th %tile	Max
Amikacin	>120 to ≤240	14.3	83.9	1.8	0.9	0.64	222.61	0	0	0.02	0.16	0.58	2.71	17.95
	>90 to ≤120	4.9	90.1	5.0	2.6	1.52	170.25	0	0.01	0.13	0.62	1.83	5.82	31.75
	>60 to ≤90	9.3	89.0	1.7	0.9	0.88	179.73	0	0	0.05	0.29	1.12	3.54	16.43
	>45 to ≤60	10.6	85.7	3.7	2.0	1.14	212.08	0	0	0.07	0.35	1.23	4.70	32.56
	>30 to ≤45	1.7	88.1	10.2	5.2	2.38	127.94	0	0.04	0.45	1.31	3.12	8.07	23.50
	≥16 to ≤30	-	-	-		-	-	-	-	-	-	-	-	-
Gentamicin	>120 to ≤240	12.8	86.2	1.0	0.7	0.45	81.62	0	0.06	0.19	0.35	0.63	1.17	2.47
	>90 to ≤120	5.0	90.0	5.0	2.9	0.73	74.05	0	0.12	0.34	0.63	0.97	1.74	3.58
	>60 to ≤90	6.1	90.9	3.0	1.6	0.67	72.03	0	0.10	0.30	0.55	0.94	1.60	3.04
	>45 to ≤60	8.3	85.3	6.4	4.0	0.78	75.61	0	0.08	0.35	0.66	1.06	1.87	3.87
	>30 to ≤45	1.6	78.5	19.9	13.4	1.23	60.13	0.01	0.23	0.70	1.11	1.61	2.65	5.24
	≥16 to ≤30	-	-	-		-	-	-	-	-	-	-	-	-
Tobramycin	>120 to ≤240	14.3	84.6	1.1	0	0.11	160.40	0	0.01	0.03	0.05	0.11	0.44	1.52
	>90 to ≤120	5.0	90.0	5.0	1.5	0.27	145.89	0.01	0.02	0.05	0.13	0.32	0.95	3.37
	>60 to ≤90	2.6	94.3	3.1	0.1	0.21	125.08	0.01	0.02	0.05	0.11	0.26	0.73	2.68
	>45 to ≤60	1.1	93.4	5.5	1.1	0.29	132.04	0.01	0.03	0.08	0.16	0.32	0.98	3.12
	>30 to ≤45	0.0	82.1	17.9	3.6	0.57	100.38	0.02	0.07	0.18	0.37	0.75	1.80	4.39
	≥16 to ≤30	-	-	-		-	-	-	-	-	-	-	-	-

a. Safety thresholds assessed for total-drug plasma C_{min} were ≥ 8 mg/L for amikacin and ≥ 2 mg/L for gentamicin and tobramycin [5, 6, 7, 8].

Table S12. Percent probabilities of PK-PD target attainment by MIC based on total-drug plasma and/or ELF AUC:MIC ratio targets associated with net bacterial stasis for Enterobacterales and *P. aeruginosa* by drug, PK-PD target selection approach, and dosing regimen among simulated patients with normal renal function, with the 2019 USCAST susceptible breakpoint shaded

Pathogen	Agent	Exposure (neutropenic murine infection model)	MIC	Percent probabilities of PK-PD target attainment by MIC based on AUC:MIC ratio targets approach associated with net bacterial stasis[a]					
				Hill-Type		Median		Randomly Assigned	
				Traditional	Extended-interval	Traditional	Extended-interval	Traditional	Extended-interval
Enterobacterales	Amikacin	Total-drug plasma (thigh)[b]	2	100	100	100	100	93.5	96.9
			4	95.4	98.7	99.9	100	80.0	84.7
			8	23.1	52.2	66.5	91.4	58.0	65.5
			16	1.00	3.90	4.80	19.3	32.0	41.1
		Total-drug ELF (lung)[c]	2	99.9	100	99.9	100	99.0	99.9
			4	90.7	97.9	90.8	98.0	79.9	90.9
			8	29.9	56.4	30.7	57.2	30.9	49.8
			16	1.60	6.30	1.70	6.60	2.40	9.30
	Gentamicin	Total-drug plasma (thigh)[b]	0.5	100	100	100	100	97.4	99.0
			1	99.8	99.8	100	100	87.6	92.2
			2	70.8	89.5	96.5	98.3	69.9	75.9
		Total-drug ELF (lung)[c]	0.5	100	100	100	100	99.9	100
			1	99.1	99.6	99.2	99.6	94.7	99.0
			2	70.5	86.6	71.0	87.0	60.3	74.5
	Tobramycin	Total-drug plasma (thigh)[b]	0.5	100	100	100	100	96.7	97.6
			1	99.1	99.5	100	100	86.1	90.0
			2	60.7	78.2	91.0	95.7	67.4	72.1
		Total-drug ELF (lung)[c]	0.5	100	100	100	100	99.9	99.8
			1	97.4	99.0	97.5	99.0	91.3	96.4
			2	61.3	77.2	62.6	77.3	52.9	65.7

Table S12. Percent probabilities of PK-PD target attainment by MIC based on total-drug plasma and/or ELF AUC:MIC ratio targets associated with net bacterial stasis for Enterobacterales and *P. aeruginosa* by drug, PK-PD target selection approach, and dosing regimen among simulated patients with normal renal function, with the 2019 USCAST susceptible breakpoint shaded

Pathogen	Agent	Exposure (neutropenic murine infection model)	MIC	Percent probabilities of PK-PD target attainment by MIC based on AUC:MIC ratio targets approach associated with net bacterial stasis[a]					
				Hill-Type		Median		Randomly Assigned	
				Traditional	Extended-interval	Traditional	Extended-interval	Traditional	Extended-interval
P. aeruginosa	Amikacin	Total-drug plasma (thigh)[d]	1	100	100	100	100	99.7	100
			2	97.0	99.1	97.9	99.2	87.7	95.8
			4	27.8	57.9	34.1	65.5	37.6	58.9
			8	1.20	4.80	1.50	6.00	2.90	12.9
			16	0.10	0.30	0.20	0.50	0.10	0.80
	Gentamicin	Total-drug plasma (thigh)[d]	0.5	99.8	100	99.9	100	98.0	99.6
			1	75.8	92.1	80.8	93.7	70.1	81.6
			2	4.60	22.9	6.30	27.5	15.8	31.4
	Tobramycin	Total-drug plasma (thigh)[d]	0.5	99.1	99.6	99.5	99.9	96.1	97.7
			1	65.7	82.1	71.6	84.2	62.2	74.0
			2	3.10	14.2	4.70	18.0	12.2	24.0

a. Shaded cells indicate the USCAST 2019 susceptible breakpoint.

b. Percent probabilities of PK-PD target attainment by MIC are shown for each bacterial reduction endpoint. The associated magnitude of the total-drug plasma AUC:MIC ratio target based on a neutropenic murine-thigh infection model for net bacterial stasis was 30.7 based on the Hill-type model developed using pooled data and 21.4 based on the median value. Total-drug plasma AUC:MIC ratio targets were randomly assigned based on an estimated log normal distribution of total-drug plasma AUC:MIC ratio targets associated with net bacterial stasis for the set of Enterobacterales isolates.

c. Percent probabilities of PK-PD target attainment by MIC are shown for each bacterial reduction endpoint. The associated magnitude of the total-drug ELF AUC:MIC ratio target based on a neutropenic murine-lung infection model for net bacterial stasis was 14.6 based on the Hill-type model developed using pooled data and 14.5 based on the median value. Total-drug ELF AUC:MIC ratio targets were randomly assigned based on an estimated log normal distribution of total-drug ELF AUC:MIC ratio targets associated with net bacterial stasis for the set of Enterobacterales isolates.

d. Percent probabilities of PK-PD target attainment by MIC are shown for each bacterial reduction endpoint. The associated magnitude of the total-drug plasma AUC:MIC ratio target for net bacterial stasis based on a neutropenic murine-thigh infection model was 58.3 based on the Hill-type model developed using pooled data and 55.4 based on the median value. Total-drug plasma AUC:MIC ratio targets were randomly assigned based on an estimated log normal distribution of total-drug plasma AUC:MIC ratio targets associated with net bacterial stasis for the set of *P. aeruginosa* isolates.

Table S13. Percent probabilities of PK-PD target attainment by MIC based on total-drug plasma and/or ELF AUC:MIC ratio targets associated with a 1-log_{10} CFU reduction from baseline for Enterobacterales and *P. aeruginosa* by drug, PK-PD target selection approach, and dosing regimen among simulated patients with normal renal function, with the 2023 USCAST susceptible breakpoint shaded

Pathogen	Agent	Exposure (neutropenic murine infection model)	MIC	Percent probabilities of PK-PD target attainment by MIC based on AUC:MIC ratio targets associated with a 1-log_{10} CFU reduction from baseline[a]					
				Hill-Type		Median		Randomly Assigned	
				Traditional	Extended-interval	Traditional	Extended-interval	Traditional	Extended-interval
Enterobacterales	Amikacin	Total-drug plasma (thigh)[b]	1	99.9	100	100	100	82.5	88.1
			2	67.5	92.0	94.6	98.4	58.8	67.7
			4	5.20	20.8	21.3	50.0	29.6	39.1
			8	0.30	1.50	1.00	3.60	8.20	16.2
			16	0	0	0.10	0.10	1.00	2.80
		Total-drug ELF (lung)[c]	1	100	100	99.9	100	99.5	99.9
			2	97.2	99.4	94.9	98.7	89.7	97.0
			4	51.0	74.7	42.1	67.2	48.1	66.0
			8	4.20	15.8	2.90	11.6	9.10	20.5
			16	0.40	1.00	0.30	0.90	0.20	1.70
	Gentamicin	Total-drug plasma (thigh)[b]	0.25	100	100	100	100	91.4	96.0
			0.5	96.9	98.3	99.8	99.8	73.0	78.1
			1	32.2	58.8	67.9	88.5	46.5	54.1
			2	0.30	2.10	3.10	16.5	17.6	26.8
		Total-drug ELF (lung)[c]	0.25	100	100	100	100	99.9	100
			0.5	99.8	99.9	99.5	99.8	98.0	99.7
			1	87.2	94.4	80.0	91.9	76.9	86.1
			2	22.3	43.1	15.9	34.7	25.6	41.9
	Tobramycin	Total-drug plasma (thigh)[b]	0.25	100	100	100	100	89.7	93.1
			0.5	91.7	96.4	98.9	99.4	69.2	75.1
			1	23.1	46.3	58.5	77.1	41.8	49.3
			2	0.20	1.90	1.80	9.90	15.3	22.0
		Total-drug ELF (lung)[c]	0.25	100	100	100	100	99.9	100
			0.5	99.1	99.8	98.5	99.7	96.1	99.0
			1	78.3	88.3	72.2	83.6	69.4	79.5
			2	17.9	34.0	12.4	26.2	21.0	33.7

Table S13. Percent probabilities of PK-PD target attainment by MIC based on total-drug plasma and/or ELF AUC:MIC ratio targets associated with a 1-log_{10} CFU reduction from baseline for Enterobacterales and *P. aeruginosa* by drug, PK-PD target selection approach, and dosing regimen among simulated patients with normal renal function, with the 2023 USCAST susceptible breakpoint shaded

Pathogen	Agent	Exposure (neutropenic murine infection model)	MIC	Percent probabilities of PK-PD target attainment by MIC based on AUC:MIC ratio targets associated with a 1-log_{10} CFU reduction from baseline[a]					
				Hill-Type		Median		Randomly Assigned	
				Traditional	Extended-interval	Traditional	Extended-interval	Traditional	Extended-interval
P. aeruginosa	Amikacin	Total-drug plasma (thigh)[d]	1	99.9	100	100	100	97.0	99.7
			2	67.9	92.3	80.2	96.4	69.4	82.1
			4	5.30	21.3	9.20	30.5	19.3	37.2
			8	0.30	1.60	0.40	2.00	1.00	5.30
			16	0	0	0.10	0.10	0	0.50
	Gentamicin	Total-drug plasma (thigh)[d]	0.25	100	100	100	100	99.7	99.9
			0.5	97.0	98.3	98.4	99.0	89.6	95.7
			1	32.8	59.0	48.3	74.7	46.6	62.4
			2	0.30	2.20	0.70	6.10	5.60	16.7
	Tobramycin	Total-drug plasma (thigh)[d]	0.25	100	100	100	100	99.1	99.8
			0.5	91.8	96.6	95.6	98.6	85.4	91.5
			1	23.5	47.1	36.6	60.0	40.4	53.2
			2	0.20	1.90	0.60	3.60	5.10	11.8

a. Shaded cells indicate the MIC value representing the USCAST 2023 susceptible breakpoint.

b. Percent probabilities of PK-PD target attainment by MIC are shown for each bacterial reduction endpoint. The associated magnitude of the non-clinical total-drug plasma AUC:MIC ratio target based on a neutropenic murine-thigh infection model for a 1-log_{10} CFU reduction from baseline was 84.3based on the Hill-type model developed using pooled data and 62.5 based on the median value. Total-drug plasma AUC:MIC ratio targets were randomly assigned based on an estimated log normal distribution of total-drug plasma AUC:MIC ratio targets associated with a 1-log_{10} CFU reduction from baseline for the set of Enterobacterales isolates.

c. Percent probabilities of PK-PD target attainment by MIC are shown for each bacterial reduction endpoint. The associated magnitude of the non-clinical total-drug ELF AUC:MIC ratio target based on a neutropenic murine-lung infection model and a 1-log_{10} CFU reduction from baseline was 23.7 based on the Hill-type model developed using pooled data and 25.7 based on the median value. Total-drug plasma AUC:MIC ratio targets were randomly assigned based on an estimated log normal distribution of total-drug plasma AUC:MIC ratio targets associated with a 1-log_{10} CFU reduction from baseline for the set of Enterobacterales isolates.

d. Percent probabilities of PK-PD target attainment by MIC are shown for each bacterial reduction endpoint. The associated magnitude of the non-clinical total-drug plasma AUC:MIC ratio target for a 1-log_{10} CFU reduction from baseline based on a neutropenic murine-thigh infection model was 83.9 based on the Hill-type model developed using pooled data and 74.3 based on the median value. Total-drug plasma AUC:MIC ratio targets were randomly assigned based on an estimated log normal distribution of total-drug plasma AUC:MIC ratio targets associated with a 1-log_{10} CFU reduction from baseline for the set of *P. aeruginosa* isolates.

Table S14. Percent probabilities of PK-PD target attainment by MIC based on total-drug plasma AUC:MIC ratio targets for Enterobacterales from a Hill-type model developed using pooled data from a neutropenic murine-thigh infection model among simulated patients by CLcr group after administration of traditional aminoglycoside dosing regimens, with the 2019 and 2023 USCAST susceptible breakpoints associated with net bacterial stasis and 1-log_{10} CFU reduction from baseline, respectively, shaded

Agent	MIC (μg/mL)	Percent probabilities of PK-PD target attainment by MIC and CLcr (mL/min) group[a]											
		Net bacterial stasis						1-log_{10} CFU reduction from baseline					
		≥ 16 to ≤ 30	> 30 to ≤ 45	> 45 to ≤ 60	> 60 to ≤ 90	> 90 to ≤ 120	> 120 to ≤ 240	≥ 16 to ≤ 30	> 30 to ≤ 45	> 45 to ≤ 60	> 60 to ≤ 90	> 90 to ≤ 120	> 120 to ≤ 240
Amikacin	1	100	100	100	100	100	100	99.0	99.9	98.9	100	99.9	99.9
	2	100	100	100	100	100	100	51.6	79.2	50.1	90.7	67.5	83.7
	4	88.8	97.6	84.9	99.1	95.4	97.9	0.30	5.80	2.30	14.5	5.20	13.2
	8	7.30	28.2	12.0	48.8	23.1	40.9	0	0	0	0.30	0.30	0.60
	16	0	0.30	0.60	2.30	1.00	2.80	0	0	0	0	0	0
Gentamicin	0.25	100	100	100	100	100	100	99.2	99.6	99.5	100	100	100
	0.5	99.8	100	100	100	100	100	87.0	91.4	81.9	92.7	96.9	97.4
	1	97.0	97.9	95.9	98.6	99.8	99.7	15.3	18.8	8.60	21.1	32.2	43.6
	2	54.6	61.6	40.7	62.5	70.8	80.2	0	0	0	0.10	0.30	0.30
Tobramycin	0.25	100	100	100	100	100	100	100	100	99.6	99.9	100	100
	0.5	100	100	100	100	100	100	95.8	94.6	82.6	87.4	91.7	93.2
	1	99.6	98.9	96.2	98.1	99.1	98.8	19.6	19.9	7.00	16.8	23.1	30.7
	2	67.6	68.8	37.8	53.2	60.7	67.5	0	0	0	0	0.20	0.40

a. Shaded cells indicate the MIC values representing the USCAST 2019 susceptible breakpoint based on the total-drug plasma AUC:MIC ratio target associated with net bacterial stasis and the 2023 USCAST susceptible breakpoint based on the total-drug plasma AUC:MIC ratio target associated with a 1-log_{10} CFU reduction from baseline.

Table S15. Percent probabilities of PK-PD target attainment by MIC based on total-drug plasma AUC:MIC ratio targets for Enterobacterales from a Hill-type model developed using pooled data from a neutropenic murine-thigh infection model among simulated patients by CLcr group after administration of extended-interval aminoglycoside dosing regimens, with the 2019 and 2023 USCAST susceptible breakpoints associated with net bacterial stasis and 1-$\log_{10}$ CFU reduction from baseline, respectively, shaded

Agent	MIC (µg/mL)	Percent probabilities of PK-PD target attainment by MIC and CLcr (mL/min) group[a]											
		Net bacterial stasis						1-$\log_{10}$ CFU reduction from baseline					
		≥ 16 to ≤ 30	> 30 to ≤ 45	> 45 to ≤ 60	> 60 to ≤ 90	> 90 to ≤ 120	> 120 to ≤ 240	≥ 16 to ≤ 30	> 30 to ≤ 45	> 45 to ≤ 60	> 60 to ≤ 90	> 90 to ≤ 120	> 120 to ≤ 240
Amikacin	1	-	100	100	100	100	100	-	100	100	100	100	99.6
	2	-	100	100	100	100	100	-	97.5	79.3	98.7	92.0	69.4
	4	-	99.8	95.8	100	98.7	92.1	-	30.2	11.3	29.9	20.8	8.00
	8	-	72.8	37.5	75.0	52.2	26.8	-	1.20	0.40	0.50	1.50	0.10
	16	-	4.80	2.10	5.10	3.90	1.70	-	0	0	0	0	0
Gentamicin	0.25	-	100	100	100	100	100	-	100	100	100	100	100
	0.5	-	100	100	100	100	100	-	99.0	95.9	100	98.3	94.4
	1	-	99.8	99.7	100	99.8	99.3	-	66.2	37.7	74.3	58.8	36.6
	2	-	92.0	73.7	95.7	89.5	71.7	-	2.20	0.60	3.10	2.10	0.30
Tobramycin	0.25	-	100	100	100	100	100	-	100	99.9	100	100	99.6
	0.5	-	100	100	100	100	99.9	-	98.5	92.7	99.3	96.4	87.0
	1	-	100	98.6	100	99.5	96.3	-	60.6	28.2	66.7	46.3	20.4
	2	-	88.7	64.3	91.7	78.2	55.2	-	1.40	0.30	2.00	1.90	0

a. Shaded cells indicate the MIC values representing the USCAST 2019 susceptible breakpoint based on the total-drug plasma AUC:MIC ratio target associated with net bacterial stasis and the 2023 USCAST susceptible breakpoint based on the total-drug plasma AUC:MIC ratio target associated with a 1-$\log_{10}$ CFU reduction from baseline.

Table S16. Percent probabilities of PK-PD target attainment by MIC based on total-drug ELF AUC:MIC ratio targets for Enterobacterales from a Hill-type model developed using pooled data from a neutropenic murine-lung infection model among simulated patients by CLcr group after administration of traditional aminoglycoside dosing regimens, with the 2019 and 2023 USCAST susceptible breakpoints associated with net bacterial stasis and 1-log_{10} CFU reduction from baseline, respectively, shaded

Agent	MIC (µg/mL)	Percent probabilities of PK-PD target attainment by MIC and CLcr (mL/min) group[a]											
		Net bacterial stasis						1-log_{10} CFU reduction from baseline					
		≥ 16 to ≤ 30	> 30 to ≤ 45	> 45 to ≤ 60	> 60 to ≤ 90	> 90 to ≤ 120	> 120 to ≤ 240	≥ 16 to ≤ 30	> 30 to ≤ 45	> 45 to ≤ 60	> 60 to ≤ 90	> 90 to ≤ 120	> 120 to ≤ 240
Amikacin	1	100	100	100	100	100	100	99.8	100	100	100	100	100
	2	98.2	100	99.7	100	99.9	100	93.7	98.4	95.4	99.4	97.2	98.7
	4	86.6	95.3	85.8	98.5	90.7	95.1	43.6	58.1	37.9	73.5	51.0	66.3
	8	22.9	33.7	20.7	52.8	29.9	44.7	0.90	4.70	3.30	12.3	4.20	8.80
	16	0.30	1.90	1.40	4.50	1.60	3.40	0	0	0.10	0.20	0.40	0.20
Gentamicin	0.25	100	100	100	100	100	100	99.9	100	99.9	100	100	100
	0.5	99.8	100	99.9	100	100	100	97.5	98.9	98.8	99.2	99.8	99.8
	1	95.2	97.3	95.6	98.0	99.1	99.5	76.1	79.4	68.2	79.9	87.2	89.6
	2	60.6	60.5	48.1	61.8	70.5	77.5	17.5	12.7	7.70	14.1	22.3	29.7
Tobramycin	0.25	100	100	100	100	100	100	100	100	100	100	100	100
	0.5	100	100	99.9	100	100	100	99.0	99.6	99.0	98.9	99.1	99.3
	1	97.3	97.9	95.9	96.7	97.4	98.8	85.8	84.6	67.6	73.8	78.3	81.8
	2	71.6	66.6	45.1	53.5	61.3	64.7	23.2	11.9	8.40	12.7	17.9	21.5

a. Shaded cells indicate the MIC values representing the USCAST 2019 susceptible breakpoint based on the total-drug ELF AUC:MIC ratio target associated with net bacterial stasis and the 2023 USCAST susceptible breakpoint based on the total-drug ELF AUC:MIC ratio target associated with a 1-log_{10} CFU reduction from baseline.

Table S17. Percent probabilities of PK-PD target attainment by MIC based on total-drug ELF AUC:MIC ratio targets for Enterobacterales from a Hill-type model developed using pooled data from a neutropenic murine-lung infection model among simulated patients by CLcr group after administration of extended-interval aminoglycoside dosing regimens, with the 2019 and 2023 USCAST susceptible breakpoints associated with net bacterial stasis and 1-$\log_{10}$ CFU reduction from baseline, respectively, shaded

Agent	MIC (µg/mL)	Percent probabilities of PK-PD target attainment by MIC and CLcr (mL/min) group[a]											
		Net bacterial stasis						1-$\log_{10}$ CFU reduction from baseline					
		≥ 16 to ≤ 30	> 30 to ≤ 45	> 45 to ≤ 60	> 60 to ≤ 90	> 90 to ≤ 120	> 120 to ≤ 240	≥ 16 to ≤ 30	> 30 to ≤ 45	> 45 to ≤ 60	> 60 to ≤ 90	> 90 to ≤ 120	> 120 to ≤ 240
Amikacin	1	-	100	100	100	100	100	-	100	100	100	100	100
	2	-	100	100	100	100	100	-	99.8	98.9	99.9	99.4	96.7
	4	-	98.5	96.2	99.9	97.9	90.5	-	87.8	65.7	88.3	74.7	53.6
	8	-	70.8	45.0	72.8	56.4	31.4	-	20.7	9.80	23.4	15.8	5.80
	16	-	10.2	4.60	8.90	6.30	2.50	-	1.00	0.50	0.40	1.00	0.10
Gentamicin	0.25	-	100	100	100	100	100	-	100	100	100	100	100
	0.5	-	100	100	100	100	100	-	100	99.9	100	99.9	99.8
	1	-	99.6	99.5	99.9	99.6	98.7	-	95.4	89.7	97.5	94.4	84.6
	2	-	88.6	75.5	92.8	86.6	70.6	-	47.5	28.4	55.8	43.1	22.9
Tobramycin	0.25	-	100	100	100	100	100	-	100	100	100	100	100
	0.5	-	100	100	100	100	99.8	-	99.9	99.3	100	99.8	98.6
	1	-	99.4	98.4	100	99.0	95.5	-	93.9	83.2	95.8	88.3	75.2
	2	-	85.3	68.3	88.6	77.2	55.1	-	43.3	20.8	48.7	34.0	14.5

a. Shaded cells indicate the MIC values representing the USCAST 2019 susceptible breakpoint based on the total-drug ELF AUC:MIC ratio target associated with net bacterial stasis and the 2023 USCAST susceptible breakpoint based on the total-drug ELF AUC:MIC ratio target associated with a 1-$\log_{10}$ CFU reduction from baseline.

Table S18. Percent probabilities of PK-PD target attainment by MIC based on total-drug plasma AUC:MIC ratio targets for *P. aeruginosa* from a Hill-type model developed using pooled data from a neutropenic murine-thigh infection model among simulated patients by CLcr group after administration of traditional aminoglycoside dosing regimens, with the 2019 and 2023 USCAST susceptible breakpoints associated with net bacterial stasis and 1-log$_{10}$ CFU reduction from baseline, respectively, shaded

Agent	MIC (µg/mL)	Percent probabilities of PK-PD target attainment by MIC and CLcr (mL/min) group[a]											
		Net bacterial stasis						1-log$_{10}$ CFU reduction from baseline					
		≥ 16 to ≤ 30	> 30 to ≤ 45	> 45 to ≤ 60	> 60 to ≤ 90	> 90 to ≤ 120	> 120 to ≤ 240	≥ 16 to ≤ 30	> 30 to ≤ 45	> 45 to ≤ 60	> 60 to ≤ 90	> 90 to ≤ 120	> 120 to ≤ 240
Amikacin	1	100	100	100	100	100	100	99.1	99.9	98.9	100	99.9	99.9
	2	91.9	98.8	87.5	99.4	97.0	98.6	52.6	79.8	50.8	91.0	67.9	84.2
	4	11.2	33.6	15.5	55.7	27.8	46.7	0.30	6.20	2.60	15.1	5.30	13.7
	8	0	0.60	0.70	2.70	1.20	3.40	0	0	0.10	0.30	0.30	0.60
	16	0	0	0	0.10	0.10	0.20	0	0	0	0	0	0
Gentamicin	0.25	99.9	100	100	100	100	100	99.2	99.6	99.5	100	100	100
	0.5	97.6	98.3	97.4	99.0	99.8	99.8	87.0	91.7	82.0	92.9	97.0	97.5
	1	59.5	68.7	48.6	68.4	75.8	83.7	15.5	19.0	8.80	21.4	32.8	44.1
	2	0.20	1.60	0.70	1.50	4.60	10.3	0	0	0	0.10	0.30	0.30
Tobramycin	0.25	100	100	100	100	100	100	100	100	99.6	99.9	100	100
	0.5	99.7	99.5	97.4	98.4	99.1	99.5	96.1	94.8	82.6	87.7	91.8	93.3
	1	73.9	74.3	44.3	58.3	65.7	72.2	20.6	20.2	7.00	17.6	23.5	31.5
	2	0.30	0.50	0.10	1.60	3.10	5.70	0	0	0	0	0.20	0.40

a. Shaded cells indicate the MIC values representing the USCAST 2019 susceptible breakpoint based on the total-drug plasma AUC:MIC ratio target associated with net bacterial stasis and the 2023 USCAST susceptible breakpoint based on the total-drug plasma AUC:MIC ratio target associated with a 1-log$_{10}$ CFU reduction from baseline.

Table S19. Percent probabilities of PK-PD target attainment by MIC based on total-drug plasma AUC:MIC ratio targets for *P. aeruginosa* from a Hill-type model developed using pooled data from a neutropenic murine-thigh infection model among simulated patients by CLcr group after administration of extended-interval aminoglycoside dosing regimens, with the 2019 and 2023 USCAST susceptible breakpoints associated with net bacterial stasis and 1-log_{10} CFU reduction from baseline, respectively, shaded

Agent	MIC (µg/mL)	Percent probabilities of PK-PD target attainment by MIC and CLcr (mL/min) group[a]											
		Net bacterial stasis						1-log_{10} CFU reduction from baseline					
		≥ 16 to ≤ 30	> 30 to ≤ 45	> 45 to ≤ 60	> 60 to ≤ 90	> 90 to ≤ 120	> 120 to ≤ 240	≥ 16 to ≤ 30	> 30 to ≤ 45	> 45 to ≤ 60	> 60 to ≤ 90	> 90 to ≤ 120	> 120 to ≤ 240
Amikacin	1	-	100	100	100	100	100	-	100	100	100	100	99.6
	2	-	100	97.3	100	99.1	95.0	-	97.7	79.7	98.8	92.3	69.8
	4	-	78.2	44.4	81.3	57.9	31.9	-	31.0	11.4	30.5	21.3	8.10
	8	-	6.70	2.20	7.40	4.80	2.00	-	1.20	0.40	0.50	1.60	0.10
	16	-	0	0.10	0	0.30	0	-	0	0	0	0	0
Gentamicin	0.25	-	100	100	100	100	100	-	100	100	100	100	100
	0.5	-	99.8	100	100	100	99.5	-	99.0	96.1	100	98.3	94.5
	1	-	93.8	77.7	97.1	92.1	77.1	-	66.7	38.3	75.1	59.0	37.4
	2	-	24.3	9.80	32.2	22.9	7.90	-	2.20	0.60	3.10	2.20	0.40
Tobramycin	0.25	-	100	100	100	100	99.9	-	100	99.9	100	100	99.7
	0.5	-	100	99.1	100	99.6	97.4	-	98.6	92.9	99.3	96.6	87.2
	1	-	91.3	69.8	93.7	82.1	60.9	-	60.7	28.5	67.3	47.1	20.8
	2	-	22.0	7.00	26.6	14.2	3.40	-	1.60	0.30	2.00	1.90	0

a. Shaded cells indicate the MIC values representing the USCAST 2019 susceptible breakpoint based on the total-drug plasma AUC:MIC ratio target associated with net bacterial stasis and the 2023 USCAST susceptible breakpoint based on the total-drug plasma AUC:MIC ratio target associated with a 1-log_{10} CFU reduction from baseline.

Table S20. Percent probabilities of PK-PD target attainment at MIC values representing 2023 USCAST susceptible breakpoints based on total-drug plasma and/or ELF AUC:MIC ratio targets associated with a 1-log_{10} CFU reduction from baseline for Enterobacterales and *P. aeruginosa* from a Hill-type model developed using pooled data from neutropenic murine-thigh and -lung models among simulated patients by CLcr group after administration of extended-interval aminoglycoside dosing regimens

Pathogen	Agent	2023 USCAST susceptible breakpoint	Neutropenic murine infection model	Percent probabilities of PK-PD target attainment by MIC and CLcr (mL/min) group[a]					
				≥ 16 to ≤ 30	> 30 to ≤ 45	> 45 to ≤ 60	> 60 to ≤ 90	> 90 to ≤ 120	> 120 to ≤ 240
Enterobacterales	Amikacin	2	thigh	-	97.5	79.3	98.7	92.0	69.4
			lung	-	99.8	98.9	99.9	99.4	96.7
	Gentamicin	0.5	thigh	-	99.0	95.9	100	98.3	94.4
			lung	-	100	99.9	100	99.9	99.8
	Tobramycin	0.5	thigh	-	98.5	92.7	99.3	96.4	87.0
			lung	-	99.9	99.3	100	99.8	98.6
P. aeruginosa	Amikacin	2	thigh	-	97.7	79.7	98.8	92.3	69.8
	Gentamicin	0.5	thigh	-	99.0	96.1	100	98.3	94.5
	Tobramycin	0.5	thigh	-	98.6	92.9	99.3	96.6	87.2

a. Shaded cells indicate probabilities of PK-PD target attainment ≥ 90%.

Supplemental Figures

Figure S1. Box-and-whisker plots of average total-drug plasma AUC_{0-24} over 48 hours among simulated patients by CLcr group after administration of traditional (A) and extended-interval (B) amikacin dosing regimens

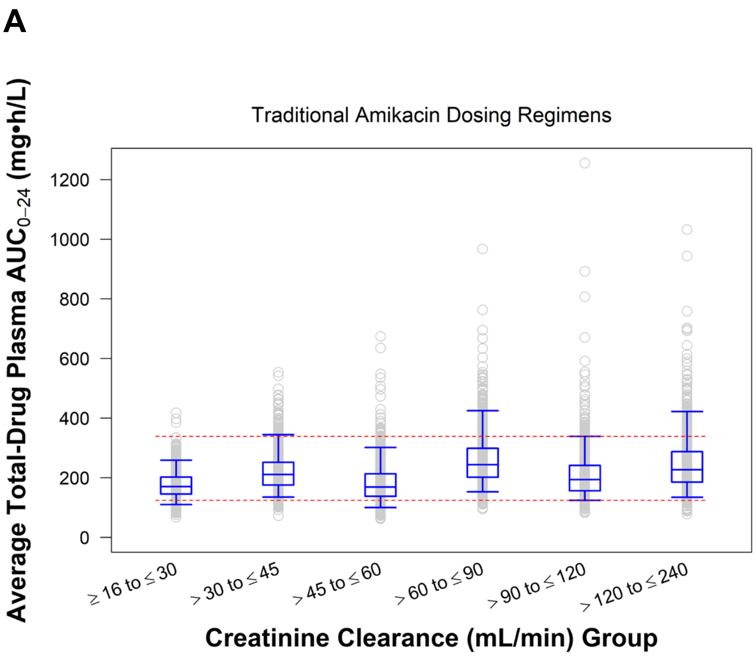

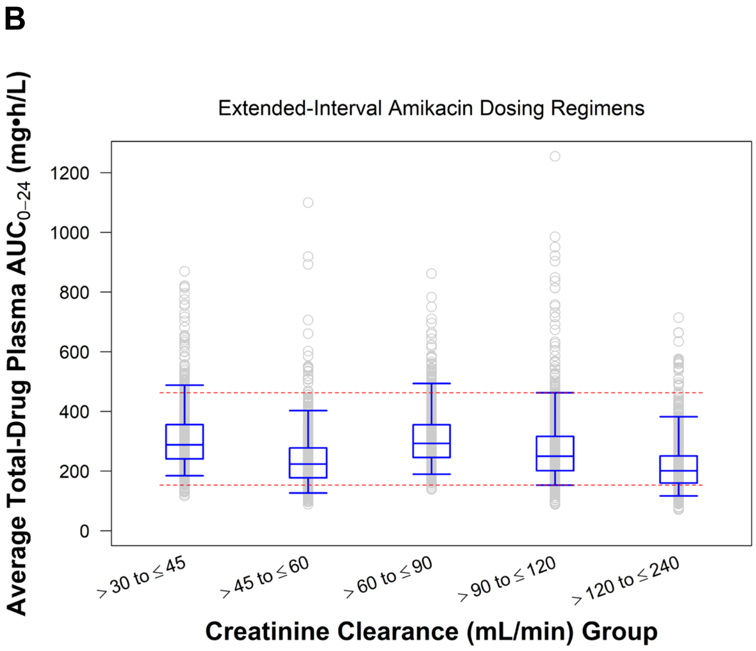

The dashed horizontal red lines represent the 90% prediction interval of the CLcr > 90 to ≤ 120 mL/min group, defined as the 5th and 95th percentiles for average amikacin total-drug plasma AUC_{0-24}.

Figure S2. Box-and-whisker plots of average total-drug plasma AUC0-24 over 48 hours among simulated patients by CLcr group after administration of traditional (A) and extended-interval (B) gentamicin dosing regimens

A

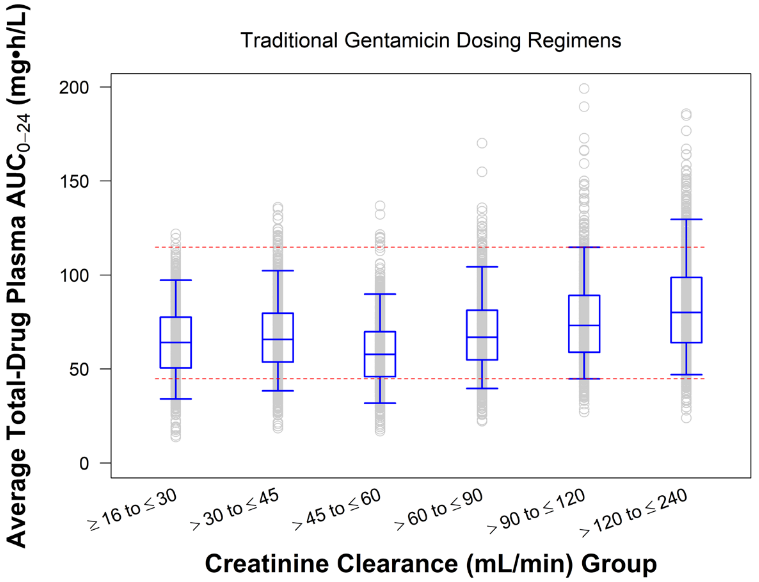

B

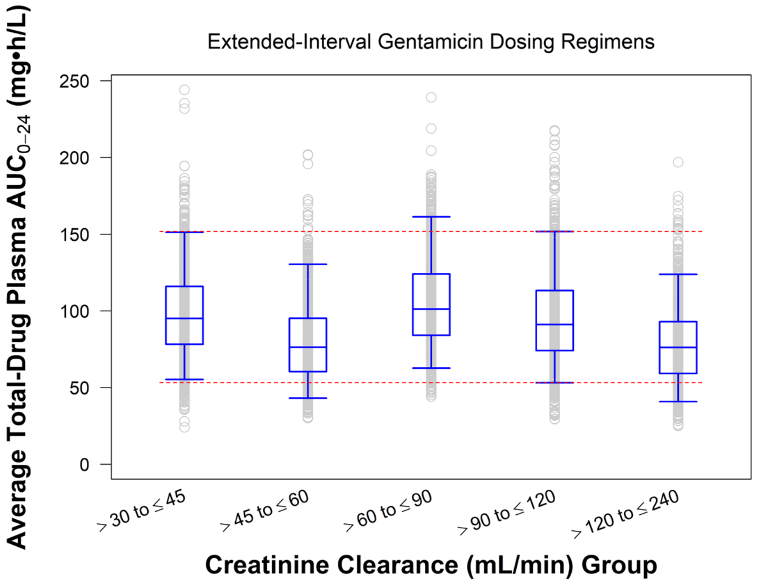

The dashed horizontal red lines represent the 90% prediction interval of the CLcr > 90 to ≤ 120 mL/min group, defined as the 5th and 95th percentiles for average gentamicin total-drug plasma AUC0-24.

Figure S3. Box-and-whisker plots of average total-drug plasma AUC_{0-24} over 48 hours among simulated patients by CLcr group after administration of traditional (A) and extended-interval (B) tobramycin dosing regimens

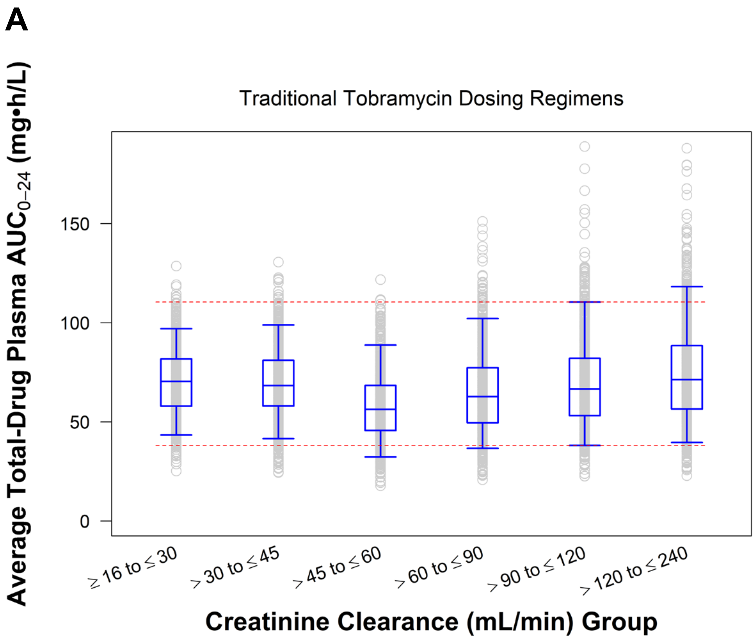

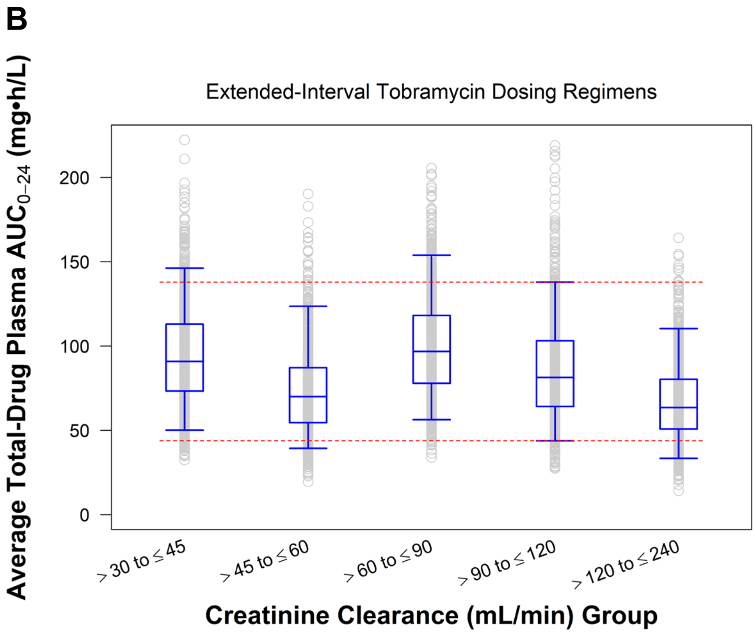

The dashed horizontal red lines represent the 90% prediction interval of the CLcr > 90 to ≤ 120 mL/min group, defined as the 5th and 95th percentiles for average tobramycin total-drug plasma AUC_{0-24}.

Figure S4. Box-and-whisker plots of average total-drug plasma C_{min} over 48 hours among simulated patients by CLcr group after administration of traditional (A) and extended-interval (B) amikacin dosing regimens

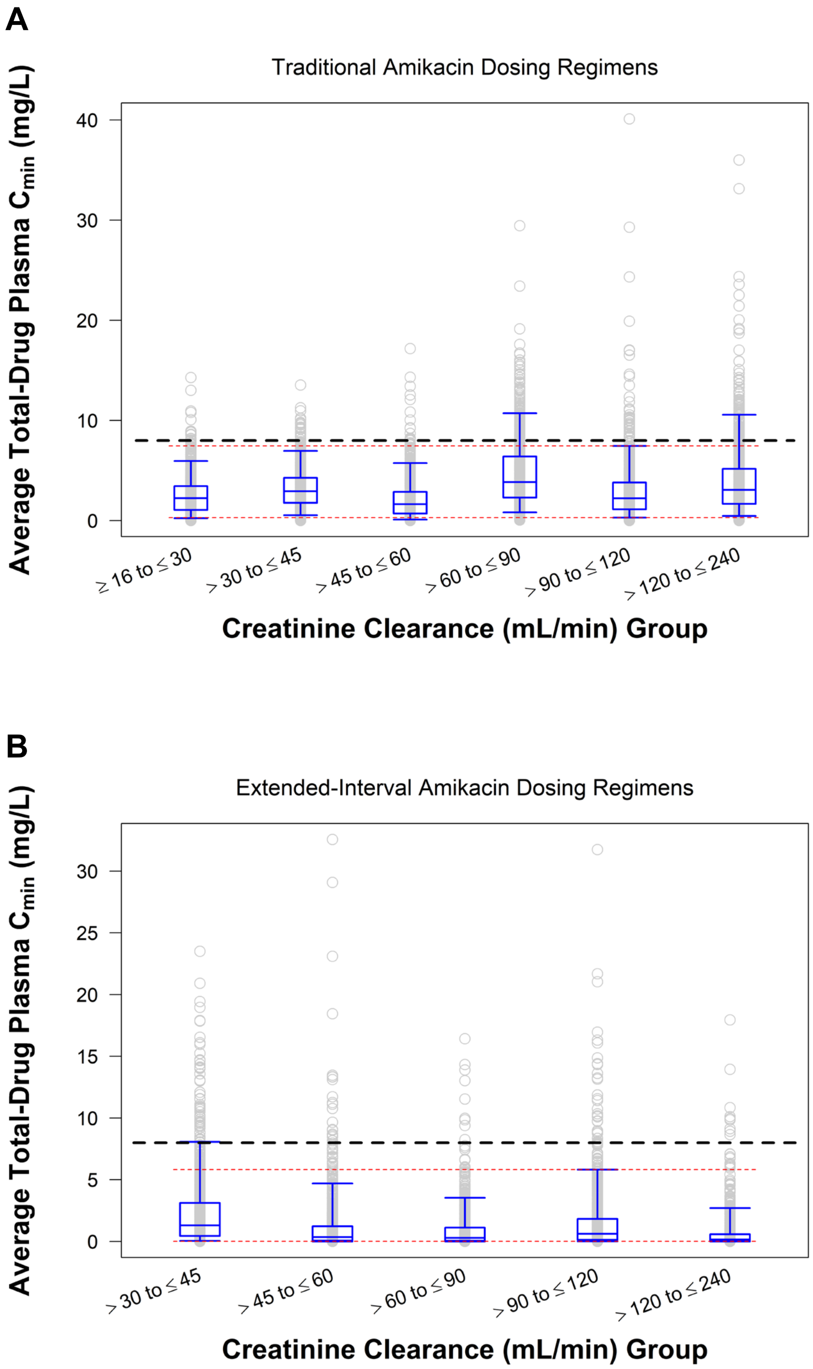

The dashed horizontal red lines represent the 90% prediction interval of the CLcr > 90 to ≤ 120 mL/min group, defined as the 5th and 95th percentiles for average amikacin total-drug plasma C_{min}. The horizontal black line represents an amikacin total-drug plasma C_{min} value of 8 mg/L.

Figure S5. Box-and-whisker plots of average total-drug plasma C_{min} over 48 hours among simulated patients by CLcr group after administration of traditional (A) and extended-interval (B) gentamicin dosing regimens

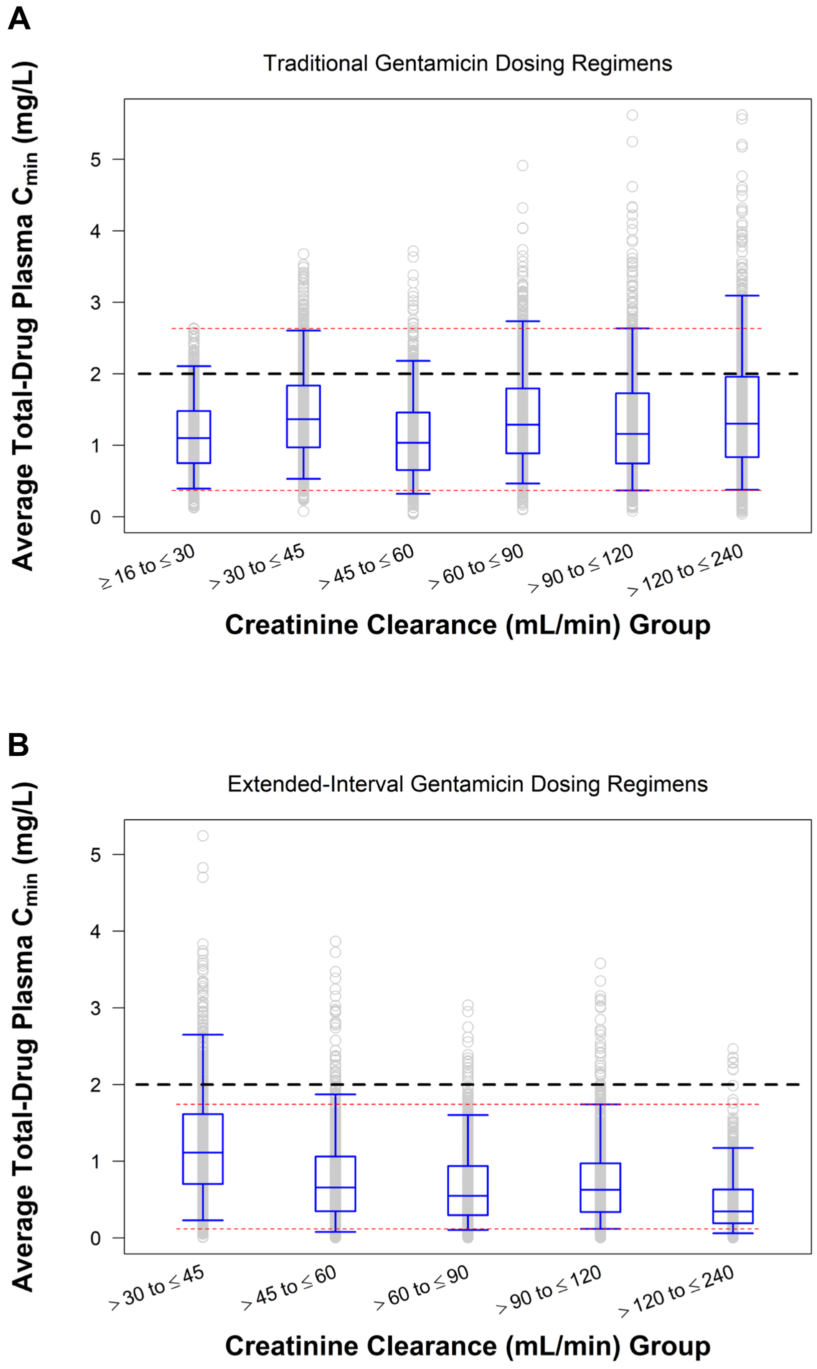

The dashed horizontal red lines represent the 90% prediction interval of the CLcr > 90 to ≤ 120 mL/min group, defined as the 5th and 95th percentiles for average gentamicin total-drug plasma C_{min}. The horizontal black line represents a gentamicin total-drug plasma C_{min} value of 2 mg/L.

Figure S6. Box-and-whisker plots of average total-drug plasma C_{min} over 48 hours among simulated patients by CLcr group after administration of traditional (A) and extended-interval (B) tobramycin dosing regimens

A

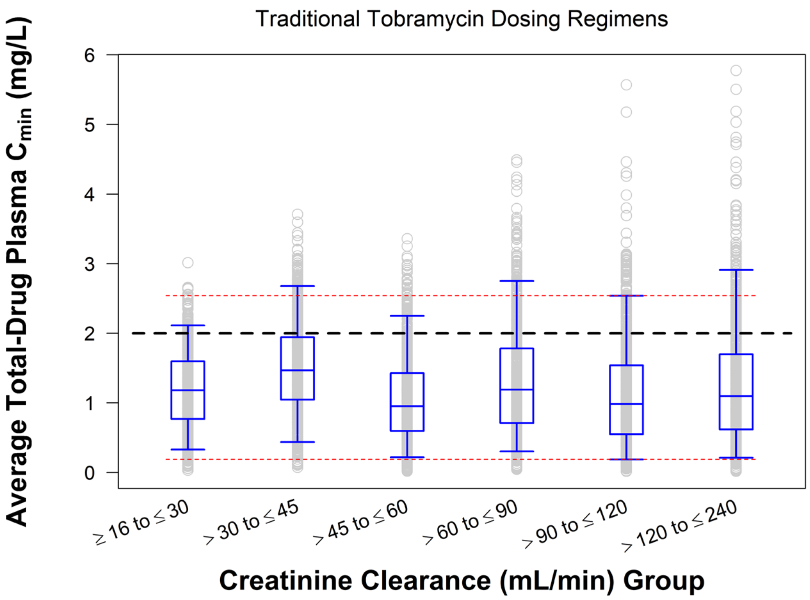

B

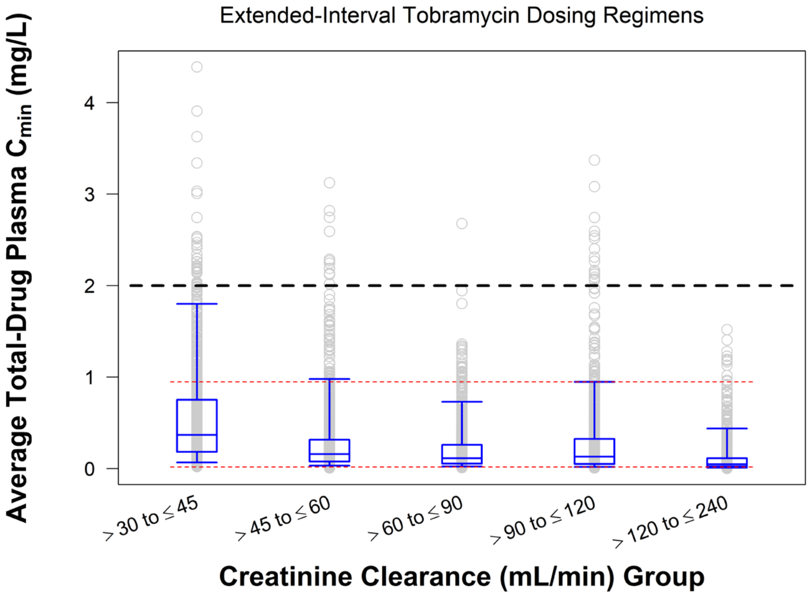

The dashed horizontal red lines represent the 90% prediction interval of the CLcr > 90 to ≤ 120 mL/min group, defined as the 5th and 95th percentiles for average tobramycin total-drug plasma C_{min}. The horizontal black line represents a tobramycin total-drug plasma C_{min} value of 2 mg/L.

Supplemental References

1. Louie A, Liu W, Fikes S, et al. Dose-response efficacy of plazomicin in the treatment of murine pneumonia due to 7 strains of *Klebsiella pneumoniae* including strains resistant to legacy aminoglycosides [Poster A-042]. Poster presented at: 52nd Interscience Conference on Antimicrobial Agents and Chemotherapy; September 9–12, 2012; San Francisco, CA, USA.
2. Louie A, Liu W, Fikes S, Brown D, Drusano GL. Impact of meropenem in combination with tobramycin in a murine model of *Pseudomonas aeruginosa* pneumonia. Antimicrob Agents Chemother. 2013;57(6):2788-2792.
3. R Development Core Team (2016) R: A language and environment for statistical computing. R. Foundation for Statistical Computing, Vienna, Austria, ISBN 3-900051-07-0.
4. Baron KT (2017). mrgsolve: Simulate from ODE-based population PK/PD and systems pharmacology models. R package Version 0.8.6.
5. Bowers DR, Schilling AN, Tam VH. Aminoglycoside Pharmacodynamics. In: Rotschafer, John C, David R. Andes, and Keith A. Rodvold, eds. Antibiotic pharmacodynamics. Springer, New York. 2016: 199-220.
6. Amikacin sulfate injection [package Insert]. 2022. Sagent Pharmaceuticals, Inc., Schaumburg, IL. Available at http://dailymed.nlm.nih.gov/dailymed/spl-resources-all-drug-labels.cfm. Accessed on April 8, 2025.
7. Gentamicin sulfate injection, solution [package Insert]. 2024. Hospira, Inc., Lake Forest IL. Available at http://dailymed.nlm.nih.gov/dailymed/spl-resources-all-drug-labels.cfm. Accessed on April 8, 2025.
8. Tobramycin sulfate injection, solution [package insert]. 2024. Hospira, Inc. Lake Forest IL. Available at http://dailymed.nlm.nih.gov/dailymed/spl-resources-all-drug-labels.cfm. Accessed on April 8, 2025.
9. Gordon RC, Regamey C, Kirby WM. Serum protein binding of the aminoglycoside antibiotics. Antimicrob Agents Chemother 1972;2(3):214-216.
10. Ramirez-Ronda CH, Holmes RK, Sanford JP. Effects of divalent cations on binding of aminoglycoside antibiotics to human serum proteins and to bacteria. Antimicrob Agents Chemother 1975;7(3):239-245.
11. Kirby WM, Clarke JT, Libke RD, Regamey C. Clinical pharmacology of amikacin and kanamycin. J Infect Dis 1976;134 SUPPL:S312-S315.
12. Rosenkranz H, Scheer M, Scholtan W. Binding of aminoglycoside antibiotics to human serum proteins. III. Effect of experimental conditions. Infection 1978;6(2):57-64.
13. Griffiths WC, Belliveau JF, Bensted CL, Calabresi SG, Diamond I. Chromatographic separation of the C(1), C(1a), and C(2) components of gentamicin and the assessment of their individual binding to serum proteins. J Pharm Sci 1984;73(12):1836-1837.

14. Carcas AJ, García-Satué JL, Zapater P, Frías-Iniesta J. Tobramycin penetration into epithelial lining fluid of patients with pneumonia. Clin Pharmacol Ther 1999;65(3):245-250.
15. Van Wart SA, Trang M, Safir MC, Santulli AR, Rubino CM, Bhavnani SM. Population pharmacokinetic analysis of tobramycin in serum and ELF using data from patients with pneumonia. Antimicrob Agents Chemother 2025; 69:e0090824.
16. The United States Committee on Antimicrobial Susceptibility Testing (USCAST). Aminoglycoside *in vitro* susceptibility test interpretive criteria evaluations. Version 1.3, February 24, 2019. https://www.uscast.org/documents. Accessed on April 8, 2025.
17. European Committee on Antimicrobial Susceptibility Testing. MIC distributions and the setting of epidemiological cut-off value (ECOFF) setting, EUCAST SOP 10.2. 2021. Available at: https://www.eucast.org/about-eucast/sops/. Accessed on April 8, 2025.
18. Kahlmeter G, Turnidge J. How to: ECOFFs-the why, the how, and the don'ts of EUCAST epidemiological cutoff values. Clin Microbiol Infect 2022;28(7):952-954.
19. Romano S, Del Mar Fdez de Gatta M, Calvo V, Mendez E, Domínguez-Gil A, Lanao JM. Influence of clinical diagnosis in the population pharmacokinetics of amikacin in intensive care unit patients. Clin Drug Investig 1998;15(5):435-444.
20. Xuan D, Nicolau DP, Nightingale CH. Population pharmacokinetics of gentamicin in hospitalized patients receiving once-daily dosing. Int J Antimicrob Agents 2004;23(3):291-295.
21. Aarons L, Vozeh S, Wenk M, Weiss P, Follath F. Population pharmacokinetics of tobramycin. Br J Clin Pharmacol 1989;28(3):305-314.
